# In vivo PAR-CLIP (viP-CLIP) of liver TIAL1 unveils targets regulating cholesterol synthesis and secretion

**DOI:** 10.1038/s41467-023-39135-8

**Published:** 2023-06-09

**Authors:** Hasan Vatandaslar, Aitor Garzia, Cindy Meyer, Svenja Godbersen, Laura T. L. Brandt, Esther Griesbach, Jeffrey A. Chao, Thomas Tuschl, Markus Stoffel

**Affiliations:** 1 Institute of Molecular Health Sciences, ETH Zurich, Otto-Stern-Weg 7, 8093 Zürich, Switzerland; 2 Laboratory of RNA Molecular Biology, The Rockefeller University, 1230 York Avenue, New York, NY 10021 USA; 3 Friedrich Miescher Institute for Biomedical Research, Maulbeerstrasse 66, 4058 Basel, Switzerland; 4 Medical Faculty, University of Zürich, 8091 Zürich, Switzerland

**Keywords:** Hepatocytes, RNA, Gene expression profiling

## Abstract

System-wide cross-linking and immunoprecipitation (CLIP) approaches have unveiled regulatory mechanisms of RNA-binding proteins (RBPs) mainly in cultured cells due to limitations in the cross-linking efficiency of tissues. Here, we describe viP-CLIP (in vivo PAR-CLIP), a method capable of identifying RBP targets in mammalian tissues, thereby facilitating the functional analysis of RBP-regulatory networks in vivo. We applied viP-CLIP to mouse livers and identified Insig2 and ApoB as prominent TIAL1 target transcripts, indicating an important role of TIAL1 in cholesterol synthesis and secretion. The functional relevance of these targets was confirmed by showing that TIAL1 influences their translation in hepatocytes. Mutant *Tial1* mice exhibit altered cholesterol synthesis, APOB secretion and plasma cholesterol levels. Our results demonstrate that viP-CLIP can identify physiologically relevant RBP targets by finding a factor implicated in the negative feedback regulation of cholesterol biosynthesis.

## Abstract (web summary)

Here the authors develop viP-CLIP, a technique allowing the identification of RNA binding proteins (RBP) targets in mouse tissues, and identify the RBP Tial1 as a regulator of cholesterol metabolism in the liver.

## Introduction

RNA-binding proteins (RBPs) interact with nascent RNA co-transcriptionally and initiate a complex process referred to as post-transcriptional gene regulation (PTGR) influencing RNA maturation, transcription, splicing, editing, subcellular localization, translation or decay^1,2^. Consequently, it is not surprising that aberrations in RBPs govern a broad spectrum of biological processes and human diseases^3^.

Several methods have been developed to identify the targets and binding sites of RBPs^4^. Most common approaches are based on cross-linking using ultraviolet (UV) light and immunoprecipitation (CLIP). The PAR-CLIP method in addition uses the photoactivatable ribonucleoside 4-thiouridine (4SU)^5^, which is incorporated into the nascent RNA transcripts of living cells. Upon UV-light excitation, the labeled RNAs yield aromatic amino acid side chain photoadducts with interacting RBPs and result in a T-to-C mutation during reverse transcription at the position of cross-linking. This mutation precisely locates the sites of RNA–RBP interaction with nucleotide resolution and enables the computational removal of ubiquitous co-purifying noncrosslinked background RNA fragments. Most CLIP methods, including PAR-CLIP, have been mainly used for the study of cells grown in monolayers and have not been adapted for in vivo applications, thereby emphasizing the need for the development of robust CLIP methods for the analysis of normal and disease tissues and organs.

The RBP ‘T-cell-restricted intracellular antigen 1 like 1’ (Tial1) belongs to the Tia1 family and has been linked to important biological processes such as tumor growth^6^, cell proliferation^7^ and apoptosis^8^. TIAL1 family proteins are ubiquitously present and are highly conserved in mammals with functional and structural homologs in other eukaryotic organisms^9,10^. They have three N-terminal RNA recognition motifs (RRMs), that were found to bind to U-rich sequences in in vitro assays^11–13^ and in PAR-CLIP studies using HEK293 cells^14^. The binding of TIAL1 family proteins to RNA modulates several aspects of RNA metabolism in the nucleus and cytoplasm. For example, TIAL1 family proteins have been shown to interact with Pol II to regulate transcription^15^, to control alternative splicing of pre-mRNA^16–18^, and to regulate the stability and translation of mRNA by binding to untranslated regions^16,18–22^. Furthermore, the C-terminal glutamine-rich prion like domain (PrLD) has been shown to be essential in stress granules formation^23^. Lastly, mice lacking *Tial1* exhibit partial embryonic lethality and defects in germ cell maturation^24^, demonstrating its importance in essential aspects of development.

Cholesterol is an essential structural component of animal cell membranes and is pivotal for lipid transport, signal transduction processes, synthesis of steroid hormones and bile salts, thereby impacting of digestion, metabolism, and endocrine function^25^. Abnormal levels of plasma cholesterol, however, can lead the development of cardiovascular disease^26^, as evidenced by high cholesterol that accumulates in arterial walls leading to the development of atherosclerosis^27^, the main cause of morbidity in Western societies^28^. Therefore, the regulation of its synthesis and secretion is a highly regulated and complex process to maintain normal cholesterol homeostasis.

The liver is the principal site responsible for the maintenance of cholesterol homeostasis by balancing the interplay of multiple pathways such as de novo synthesis, uptake and secretion of cholesterol and bile acids, lipoprotein synthesis, and reverse cholesterol transport (RTC). Cholesterol is primarily synthesized from acetyl-CoA through the mevalonate pathway. Insulin-induced gene 1 (INSIG1) and −2 (INSIG2) are ER membrane-embedded proteins that act as key negative regulators of the cholesterol biosynthesis pathway^29^. INSIG proteins can be retained by sterol-regulatory element-binding proteins (SREBPs) at the ER, blocking their processing to their active nuclear form. The nuclear SREBPs are positive transcriptional regulators of fatty acid and cholesterol synthesis, including the rate-limiting enzyme HMG-CoA reductase (HMGCR)^29,30^, which is also a common target for the cholesterol-lowering drug class of statins^31^. Furthermore, INSIG proteins can also promote ubiquitination and proteasomal degradation of HMGCR^32^.

Apolipoprotein B-100 (APOB-100) is a critical component for the packaging and secretion of the lipoprotein particles to maintain hepatic lipid homeostasis. Defects in APOB metabolism are linked to nonalcoholic fatty liver disease (NAFLD) and cardiovascular disease, demonstrating its importance in lipid homeostasis^33^. Over the last three decades, research has shown regulation of apoB production on multiple levels including transcription-factors^34,35^, post-transcriptional regulation by RBPs and miRNAs^36–38^. Moreover, co- and post-translational degradation of APOB100 during the assembly process also contribute to the in vivo regulation of apoB secretion in mammals^39,40^.

In the present study, we employed our developed viP-CLIP method and identified TIAL1 mRNA targets in the liver of mice providing insights into the metabolic regulation of lipid metabolism. We demonstrated that TIAL1 associated with the transcripts of *Insig2* and *ApoB*, thereby regulating de novo cholesterol synthesis and transport via apolipoproteins. Furthermore, we confirmed the relevance of these findings in conditional hepatocyte-specific *Tial1* knockout mouse and in adenoviral Tial1 overexpression models. The viP-CLIP-identified TIAL1 targets in liver were dysregulated upon *Tial1* ablation or overexpression, leading to imbalances in cholesterol homeostasis. These studies identified a novel posttranscriptional regulator of cholesterol metabolism.

## Results

### Development of viP-CLIP to characterize mRNA targets of RBPs in mice

**Fig. 1:**
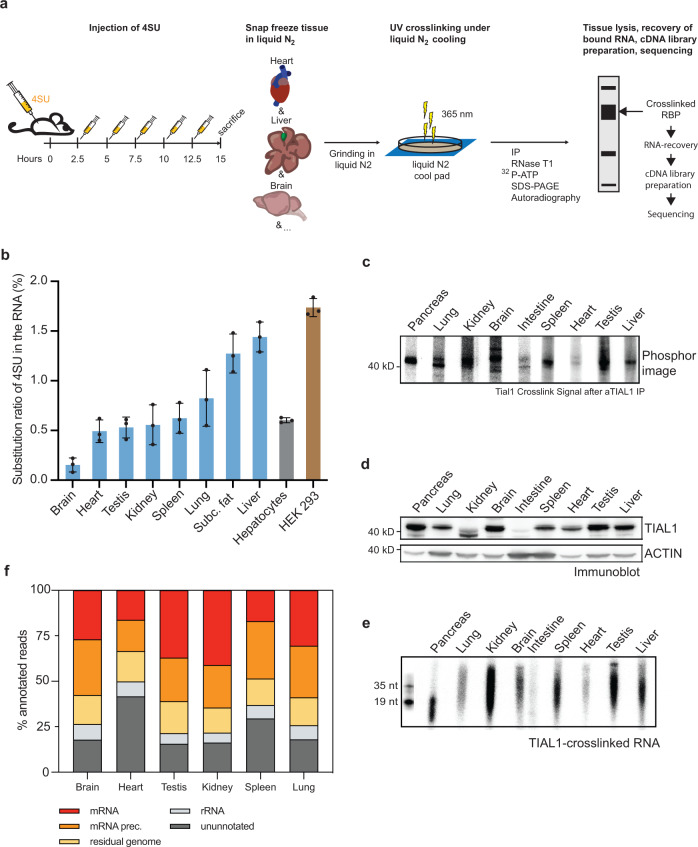
A method for determination of RBP-RNA interaction in vivo in mice. **a** Schematic representation of in vivo PAR-CLIP (viP-CLIP) applied in mice. Mice are injected 6 times with 4SU over the time course of 12.5 h and sacrificed 15 h after the first dose. Intact organs are flash-frozen, grinded, and cross-linked with UV light (365 nm) under liquid nitrogen cooling. After cell lysis of the tissue powder, the RNA-RBP complexes are immunoprecipitated in presence of RNase, radiolabeled and the recovered RNA in the size of 19–35 nt is ligated to sequencing adapter, and used for cDNA library preparation and sequencing. **b** 4SU incorporation rates in tissues (*n* = 3) after injection regime displayed in (**a**). HEK293 (*n* = 3) and primary hepatocytes (*n* = 3) were cultured in the presence of 100 mM 4SU for 16 h and subjected together with the tissue samples to HPLC analysis of 4SU incorporation rates. Values are mean +/– S.D. **c** Phosphorimage of urea-PAGE transferred to a nitrocellulose membrane that resolved ^32^P-labeled RNA-TIAL1 complexes after immunoprecipitation of TIAL1 in several tissues. **d** Immunoblot showing tissue distribution of TIAL1 in mouse organs. **e** Autoradiograph of recovered RNAs as displayed in (**c**), after proteinase K treatment and 15% urea gel electrophoresis. 5’ radiolabeled synthetic RNAs of 19 and 35 nt length served as size markers. **c**, **d**, **e** Representative images of three independent replicates. **f** Graphic presentation of viP-CLIP cDNA library composition by RNA categories from recovered RNA from (**e**). Source data are provided as a Source Data file.

Low crosslinking efficiency of RNA and protein in tissues is due to the limited penetration depth of short wavelength (254 nm) UV-light restricting RBP-RNA crosslinking mainly to single cell layers or tissue surfaces. To overcome this limitation and identify the RBP-RNA interactions in various tissues of interest (i.e., liver, brain, testis, heat, kidney, spleen) we investigated if intraperitoneal (i.p.) injections of 4SU in mice would achieve sufficient incorporation into RNA for enhanced crosslinking (Fig. 1a). We first established a method for measurement of 4SU in serum by HPLC analysis (Supplementary Fig. 1a, b). Pharmacokinetic studies indicated that 4SU peaked at 30 min in serum and remained detectable in the blood for ≈2 h after a single injection (780 mg/kg body weight) (Supplementary Fig. 1c). Furthermore, 4SU was stable in serum over time at body temperature (37 °C) (Supplementary Fig. 1d). We then evaluated the uptake and incorporation of 4SU into tissues by i.p. injecting mice every 2.5 h for 15 h, a period that covered the lifetime of most mRNAs^37^. Serum ALT, LDH and AST levels were similar in 4SU and control PBS injected mice, indicating that 4SU administration at these doses and duration was not hepatotoxic (Supplementary Fig. 1e–g). 2.5 h after the last injection, the organs were harvested, flash frozen and grinded to a fine power under liquid nitrogen. Subsequently, the powder was exposed to UV light of 365 nm at a dose of 0.3 J/cm^2^ for 4 times under continuous cooling with liquid nitrogen (Fig. 1a). We also applied this method to test crosslinking efficiency of the RBP ELAFL1 (HuR) by comparing conventional UV 254 to UV 365 nm crosslinking. The latter improved the RNA recovery in liver tissue several fold and reached comparable levels to crosslinking experiments in monolayer cell culture (Supplementary Fig. 1h)^41^.

Next, we quantified the 4SU incorporation rates into newly synthesized mRNA of several tissues and compared the values to primary mouse hepatocytes and HEK293 cells that were grown in monolayers. Lysates were prepared in NP40 buffer of up to 2% (v:v) to efficiently lyse tissues with high-fat content (i.e., liver, brain). After lysis, the concentration of NP40 was decreased from 2% to 0.06% for efficient immunoprecipitation, as some antibodies used in our study, including Tial1, lost their binding capacity in high detergent concentrations (Supplementary Fig. 1i). Subsequently the lysate was cleared, RNaseT1 treated, and the protein of interest with its partially digested crosslinked RNA immunoprecipitated. The crosslinked target RNAs were radioactively labeled to visualize the RBP-RNA adduct after SDS gel separation and nitrocellulose membrane transfer (Fig. 1c). Tissues with low expression of the protein (e.g., the intestine) only displayed a weak crosslinking band (Fig. 1d). The RNA fragments between 19 and 40 nt were recovered and used for 3’ and 5’ adapter ligation and cDNA library preparation and sequencing, as in a regular PAR-CLIP protocol (Fig. 1e)^5^. Efficient recovery of crosslinking mRNA and pre-mRNA segments was revealed by identifying predominantly error distance 1 (d1) sequencing reads carrying the characteristic T-to-C transitions for all major organs, including brain, lung, kidneys and testes (Supplementary Fig. 1j, Supplementary Table 1). The crosslinked read distribution of TIAL1 to its targets was similar across several organs (Fig. 1f). The method was also reproducible for another tested RBP (HuR) in liver and adipose tissue (Supplementary Fig. 1h, k). Taken together, the viP-CLIP protocol (Supplementary Method) is a useful expansion to the conventional CLIP protocols, enabling the identification of crosslinked RNA targets from intact mouse organs with high reproducibility, thereby enabling in depth studies of RBP-RNA interactions in genetic mouse models and under diverse environmental conditions (a detailed step by step protocol is available in Protocol Exchange (https://protocolexchange.researchsquare.com/).

### Hepatic Tial1 viP-CLIP in mouse liver revealed targets involved in lipid metabolism

**Fig. 2:**
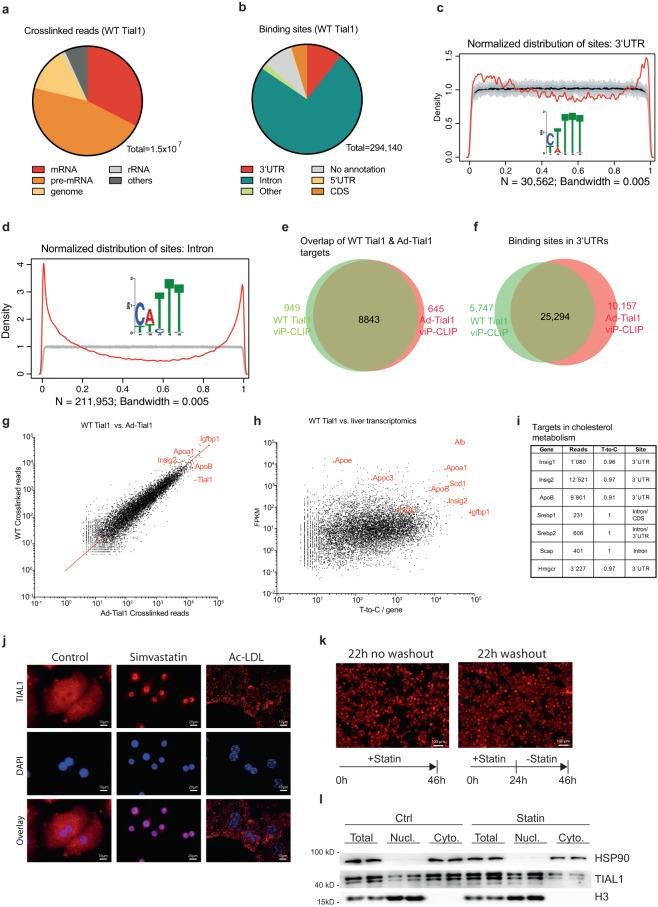
TIAL1 crosslinks and binding to target mRNAs. **a** Crosslinked Tial1 liver viP-CLIP reads harboring characteristic T-to-C conversions primarily map to the precursor mRNA and mRNA categories. **b** PARalyzer-based binding sites distribution in various RNA-species primarily indicates intronic and 3’UTR binding. **c** Normalized density distribution of Tial1 liver viP-CLIP binding sites (red line) over 3’UTRs compared to a randomized background (gray line). Tial1 binding accumulates close to the poly adenylation and cleave sites. Sequence logo for the RNA recognition element of Tial1 top 1000 3’UTR sequence read clusters is indicated in the graph. **d** Normalized density distribution of TIAL1 liver viP-CLIP binding sites (red line) over introns compared to a randomized background (gray line). An enrichment of TIAL1 binding at 5′ and 3′ splice sites is observed. Sequence logo for the RNA recognition element of Tial1 top 1000 intronic sequence read clusters is indicated in the graph. **e** Venn diagram with a target overlap of the two liver viP-CLIPs for the endogenous TIAL1 and Ad-Tial1 (*n* = 8 and *n* = 5 mice). **f** Venn diagram with an overlap of binding sites in 3’UTR for both replicates (*n* = 8 and *n* = 5 mice). **g** Scatterplot of normalized crosslinked read counts for overlapping targets of both replicates. Spearman correlation and statistics are depicted. **h** Scatterplot of T-to-C counts in crosslinked read per gene versus the expression value in FPKM. Spearman correlation and statistics are depicted. **i** Liver Tial1 viP-CLIP targets identified in the study. **j** Representative images of TIAL1 stainings in primary human hepatocytes treated with DMSO (vehicle control), AcLDL (120 µM), or Simvastatin (5 µM) for 24 h (*n* = 3). **k** Comparisons of cells treated for 46 h with statin (left) and 24 h with statin and then washed and cultured for additional 22 h without statin (*n* = 3). **l** TIAL1 immunoblot of the samples in (**j**) after nucleocytoplasmic fractionation (*n* = 2). Source data are provided as a Source Data file.

We next aimed to identify RNA targets of endogenous and overexpressed TIAL1 in livers using viP-CLIP. TIAL1 overexpression was achieved through injection of a recombinant adenovirus expressing an epitope-tagged Tial1 (Ad-Tial1) and resulted in a ≈ 2.5-fold increase in TIAL1 protein expression (Supplementary Fig. 2a). viP-CLIP was performed on three mice each using either antibodies recognizing the endogenous TIAL1 protein or an anti-myc antibody in mice infected with a recombinant adenovirus expressing a tagged TIAL1 protein (Ad-Tial1). We obtained 1.3×10^8^ sequence reads for the endogenous TIAL1 and 8.6×10^7^ for Ad-Tial1 mapping to the mm10 mouse reference transcriptome. Between 12% and 19% of the annotated reads showed the characteristic T-to-C conversion and predominantly mapped to mature and precursor mRNAs (Fig. 2a, Supplementary Fig. 2b). Binding sites with T-to-C conversions were identified by PARalyzer, resulting in 294,140 and 368,715 binding sites distributed along 9,792 and 9,488 target RNAs, respectively, for endogenous TIAL1 and overexpressed Ad-Tial1 (Fig. 2b, Supplementary Fig. 2c). Consistent with the nucleo-cytoplasmic localization of TIAL1 and complete lysis of the nucleus, 31,054 and 39,467 binding sites (11% and 11%) were found in 3’UTRs and 217,474 and 280,646 binding sites (74% and 76%) in introns (Fig. 2b, Supplementary Fig. 2d).

3’ UTR binding sites were enriched near cleavage and polyadenylation sites, indicative for functions in degradation and translational control of target transcripts (Fig. 2c, Supplementary Fig. 2e). Intronic binding sites were proximal to 5’ as well as 3’ splice sites (SSs) (Fig. 2d, Supplementary Fig. 2d), supporting an additional role in pre-mRNA splicing^16,42^. Motif analysis of 3’UTR and introns revealed a U-rich RNA recognition element (RRE) of TIAL1^14^, consistent with the previously studied RRE using electrophoretic mobility shift assays (EMSA) with purified human TIAL1 protein^14^. Both PAR-CLIP experiments resulted in similar target composition (Fig. 2e) and binding site distribution (Fig. 2f), indicating that 2.5-fold overexpression of the epitope-tagged version of TIAL1 neither alters its binding abilities nor its target make-up.

Many AU-rich element (ARE)-specific mRBPs are known to regulate mRNA stability^43^. Accordingly, we could detect a slight shift of crosslinked target reads to the endogenous TIAL1 in viP-CLIP, probably due to target destabilization, in livers of mice injected with Ad-Tial1 compared to controls (Fig. 2g). The binding sites of wildtype and overexpressed epitope-tagged TIAL1 viP-CLIP in liver and other organs are consistent with the previously studied RRE using electrophoretic mobility shift assays (EMSA) with purified human TIAL1 protein^18^.

To identify prominent transcripts that bind to TIAL1 in vivo we correlated normalized T-to-C converted reads from TIAL1 viP-CLIP to the transcriptomic data of WT mice accessible at NCBI SRA with accession number PRJNA530736 (Fig. 2h). As expected, albumin (Alb) as the highest expressed target harboring poly-U binding sites, captures many reads with T-to-C conversion (Fig. 2h). Interestingly, RNA sequencing of livers from *Tial1* liver-specific knockout (LKO) and control animals only revealed a small number of differentially expressed genes, of which only 39 overlapped with PAR-CLIP targets (Supplementary Fig. 2g). Importantly, most mRNAs enriched in T-to-C conversions belonged to lipid metabolism (Fig. 2i), as also verified by IPA pathway analysis (Supplementary Fig. 2h). Of note, two key regulators of cholesterol synthesis and lipoprotein secretion, Insig2 (CLIP rank 37, 3’UTR) and ApoB (CLIP rank 56, CDS/3’UTR), respectively, ranked among the top Tial1 targets.

A previous study reported that TIAL1 is a shuttling protein whose nuclear import is coupled to transcription^44^. No physiological stimuli have been identified that influence nuclear/cytoplasmic localization. Since TIAL1 target were enriched for lipid metabolism transcripts we explored if intracellular hepatic sterol levels affect TIAL1 expression or subcellular localization by increasing or depleting sterol pools in primary human hepatocytes. In untreated hepatocytes, TIAL1 distributed over cytoplasm and nucleus, with a slight preference for the nucleus (Fig. 2j and Supplementary Fig. 2i). Treatment of the cells with Ac-LDL for 24 h shifted the preferential nuclear localization of TIAL1 to the cytoplasm. In contrast, sterol depletion using the HMGCoA inhibitor simvastatin induced a translocation of TIAL1 into the nucleus (Fig. 2j and Supplementary Fig. 2i). Removal of simvastatin from the media reversed this effect through nuclear export of TIAL1 and relocation into the cytoplasm (Fig. 2k, Supplementary Fig. 2j). The nucleo/cytoplasmic shuttling of TIAL1 under these conditions was confirmed biochemically by subcellular fractionation and immunoblotting (Fig. 2l). However, the intracellular localization of Insig2 or ApoB transcripts did not mirror that of TIAL1 protein in cytosolic/nuclear fractions (Supplementary Fig. 2k, l) and in single-molecule fluorescent in situ hybridization (FISH) experiments (Supplementary Fig. 2m). These results indicate that TIAL1 changes cellular compartmentalization in response to intracellular cholesterol availability but that this shuttling activity does not have a major impact on the subcellular distribution of Insig2 and ApoB transcripts.

### Hepatic Tial1 regulates cholesterol synthesis through the Insig/Srebp pathway

**Fig. 3:**
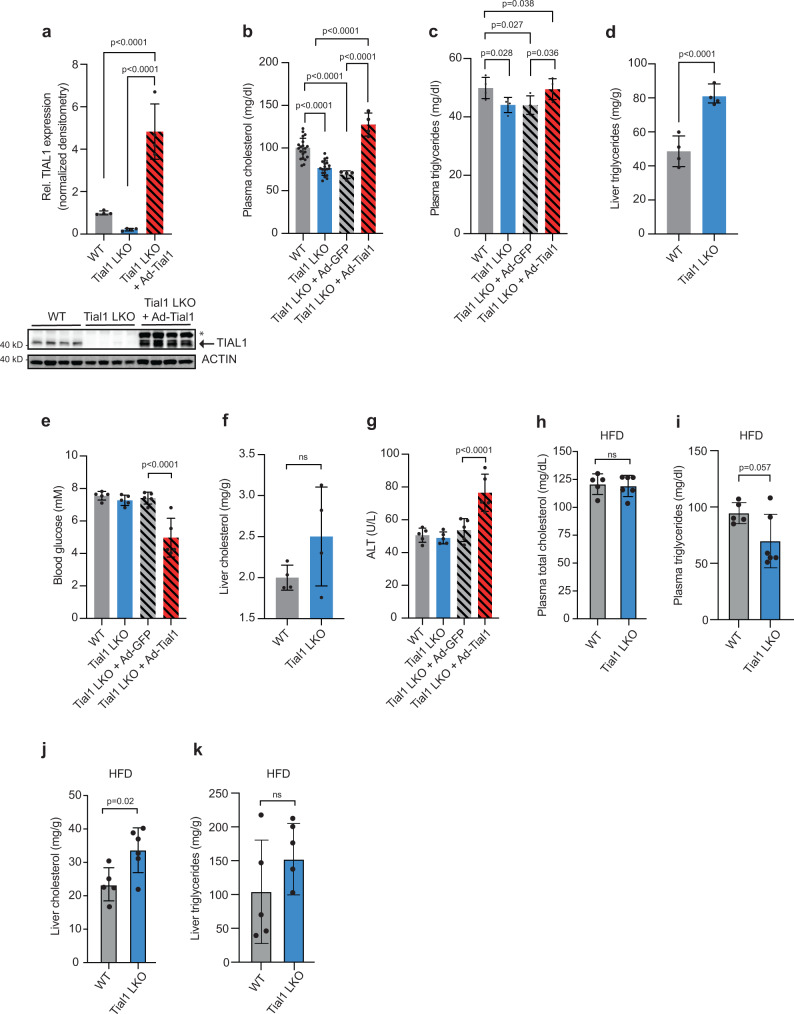
Genetic gain- and loss of function mutations in *Tial1* affect cholesterol metabolism. **a** Relative protein expression of TIAL1 in livers of wildtype (WT), Tial1 LKO and Ad-Tial1 injected Tial1 LKO animals (*n* = 4 mice per group). Values are relative densitometric readouts normalized to ACTIN. **b** Plasma total cholesterol (*n* = 20 mice for WT, *n* = 19 mice for Tial1 LKO), **c** plasma triglycerides, **d** liver triglycerides, **e** random blood glucose levels, **f** liver cholesterol, **g** alanine transaminase (ALT) levels of indicated groups fed a chow diet (*n* = 4 mice per group in **a**, **b**, **c**, **d**, **f**; *n* = 5 mice per group in **e**, **g**). Measurements of high fat diet (HFD) mice of indicated genotype of plasma cholesterol (**h**), plasma triglycerides (**i**), liver cholesterol (**j**) and liver triglycerides (**k**). Tial1LKO, and Ad-Tial1 and Ad-GFP injected Tial1 LKO animals (*n* = 5 per group). Data are presented as mean values ± SDs. Statistical significance was evaluated by two-tailed Student’s *t*-test (in **d**, **f**, **h**–**k**) or two-tailed ANOVA with Holm-Šídâk post hoc analysis (in **a**, **b**, **c**, **e**, **g**). Source data are provided as a Source Data file.

Our findings that intracellular TIAL1 distribution is regulated in response to intracellular cholesterol availability and that TIAL1 prominently binds to transcripts involved in lipid metabolism in vivo motivated us to further investigate the physiological function of TIAL1 in hepatic metabolism. We generated a liver-specific knockout by breeding albumin-Cre *(Alb-Cre)* transgenic mice with conditional Tial1KO mice (*Tial1*^*fl/fl*^), leading to a hepatocyte-specific deletion of the *Tial1* gene (Supplementary Fig. 3a, b). TIAL1 protein levels were >90% reduced in whole-cell liver extracts isolated from 8-week-old *Tial1*^*fl/fl*^ *Alb-Cre* (*Tial1 LKO*) compared to *Tial1*^*fl/fl*^ controls (*WT*) (Fig. 3a). Adenoviral Tial1 re-expression in Tial1 LKO mice resulted in a ≈ 5 to 7-fold hepatic overexpression of Tial1 mRNA and protein compared to wildtype mice (Fig. 3a, Supplementary Fig. 3b). Heterozygous (*Tial1*^*fl/+*^ *Alb-Cre, Tial1*^*+/–*^ *LKO)* and homozygous Tial1 LKO mice exhibited a 13% and 30% decrease in plasma cholesterol levels compared to WT littermates, respectively (Fig. 3b, Supplementary Fig. 3c, d). Re-expression of Tial1 through injection of Ad-Tial1 in Tial1 LKO animals restored/increased the plasma cholesterol levels to ~170%, when compared to Ad-GFP injected littermates (Fig. 3b). Plasma triglyceride levels showed the same tendency as cholesterol (Fig. 3c). Furthermore, hepatic triglyceride levels were increased ≈40% in Tial1 LKO vs control mice (Fig. 3d). No changes were measured in body weight, blood glucose, glucose tolerance, plasma free fatty acids and liver cholesterol in Tial1 LKO vs WT mice (Supplementary Fig. 3e–h, Fig. 3e, f). Notably, in mice fed a high-fat diet (HFD) plasma and liver cholesterol and triglyceride levels were similarly affected than under chow diet (Fig. 3h–k), whereas blood glucose levels, glucose tolerance and free fatty acids (FFA) were similar in *Tial1* mutant and WT mice (Supplementary Fig. 3g–l). Furthermore, no changes in plasma ALT levels were detected for the Tial1 LKO mice under chow and HFD conditions, indicating that *Tial1* ablation does not cause hepatotoxicity (Fig. 3g, Supplementary Fig. 3m). However, overexpression of Tial1 showed slightly increased ALT and decreased glucose levels (Fig. 3e, g).

**Fig. 4:**
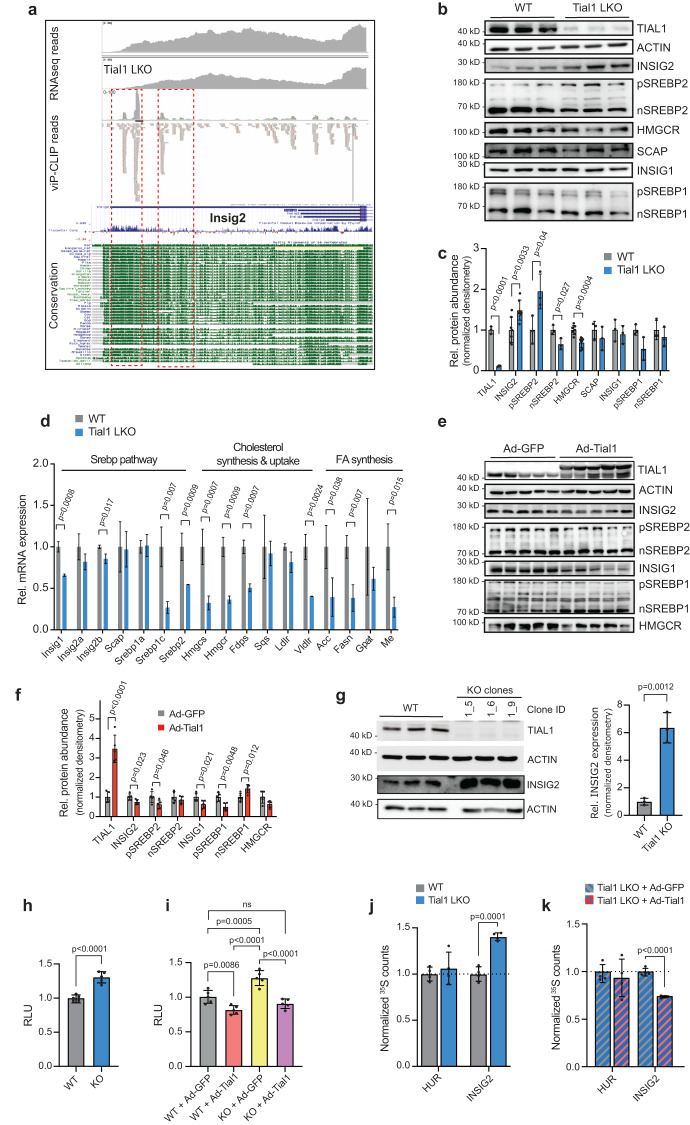
Hepatic ablation of *Tial1* reduces cholesterol biosynthesis through modulating Insig2 translation. **a** TIAL1 liver viP-CLIP reads and cluster for *Insig2* 3’UTR region, aligned to UCSC genome and conservation tracks. Section of strong binding marked in red boxes. **b** Western blot analysis of TIAL and proteins involved in the INSIG/SREBP pathway in livers of wildtype (WT) and Tial1 LKO mice. **c** Densitometric analysis of the immunoblots in (**b**) (*n* = 3). Additional samples (*n* = 4) to improve statistical power for HMGCR and INSIG2 are shown in Supplementary Fig. 4a. **d** qRT-PCR analysis of transcripts involved in Srebp pathway, cholesterol synthesis and uptake, and fatty acid synthesis (*n* = 3). **e** Western blot analysis of TIAL1 and proteins involved in the INSIG/SREBP pathway in the livers of mice injected with Ad-GFP or Ad-Tial1 (*n* = 5). **f** Densitometric analysis of the immunoblots in (**e**) (*n* = 5). **g** Relative expression of TIAL1 and INSIG2 in three independent Hepa 1–6 Tial1 knockout cell lines. Densitometric analysis of relative INSIG2 immunoblot signals of WT and Tial1 KO cell lines, normalized to ACTIN, is shown on the right. **h** 3’UTR activity of *Insig2* mRNA harboring TIAL1 binding sites in Hepa1–6 wildtype (WT) and KO cells. Data are expressed as relative luciferase activity (RLU) in WT cells transfected with psiCheck2 vector containing *Insig2* 3’UTR, normalized to 1 (*n* = 5). **i** 3’UTR activity of *Insig2* mRNA harboring TIAL1 binding sites in WT and Tial1 LKO hepatocytes, infected with Ad-GFP or Ad-Tial1. Data are expressed as relative luciferase activity normalized to WT cells transfected with Ad-GFP and psiCheck2 vector containing Insig2 3’UTR, normalized to 1 (*n* = 5). **j** ^35^S counts from metabolic labeling and immunoprecipitation of TIAL1 target INSIG2 and non-target HUR in primary mouse hepatocytes from wildtype (WT) and Tial1 LKO mice (*n* = 4). **k** ^35^S counts from metabolic labeling if HUR and INSIG2 in Tial1LKO hepatocytes that were infected with control Ad-GFP or Ad-Tial1 (*n* = 4). Data are presented as mean values ± SDs. Statistical significance was evaluated by two-tailed Student’s *t*-test (in **c**, **d**, **f**, **g**, **h**, **j**, **k**) or one-way ANOVA with Holm-Šídâk post hoc analysis (in i). Source data are provided as a Source Data file.

We next asked whether the decrease of circulating cholesterol was a consequence of reduced de novo cholesterol synthesis. The viP-CLIP analysis revealed that TIAL1 in the liver binds to the 3’UTR of Insig2. Strikingly, the binding sites defined by clustered reads mapped in regions at the 3’UTR that are highly conserved from armadillo to human, indicating important functional and regulatory roles of these locations (Fig. 4a). In this regard it is worth noting that RBPs bound to 3’UTRs are known to influence the fate of target transcripts regarding translation efficiency, stability, location or alternative polyadenylation of the 3’UTR^45^. Indeed, we found that INSIG2 protein is >2-fold upregulated in livers of Tial1 LKO animals (Fig. 4b, c), while we did not measure significant changes in steady-state cognate transcript levels (Fig. 4d). Upregulated INSIG2 retained the precursor form pSREBP2 at the ER membrane, where it was protected from cleavage to its active nuclear (nSREBP2) form. Genetic *Tial1* ablation did not affect INSIG1, SREBP1 and SCAP levels (Fig. 4b). Overall, the SREBP downstream targets Hmgcr, Hmgcs, and Fdps were reduced 50 to 65% (Fig. 4d). Furthermore, transcript levels of fatty acid synthesis, including Acc, Fasn, Gpat and Me, were also downregulated in Tial1 LKO compared to control mice and in concordance with decreased SREBP pathway activation (Fig. 4d). Adenoviral overexpression of Tial1 in WT animals had the opposite effect on target protein as well as transcript levels and displayed reduced hepatic INSIG2 protein abundance and expression compared to Ad-GFP injected animals (Fig. 4e, f, Supplementary Fig. 4b). Together, these results suggest that hepatic TIAL1 is a negative regulator of *Insig2* expression leading to reduced SREBP2 processing, decreased expression of SREBP2 downstream target genes and ultimately reduced cholesterol biosynthesis.

To gain mechanistic insights into this association we generated Hepa1–6 *Tial1* CRISPR-Cas9 knockout cells (Fig. 4g), to avoid recurring siRNA transfections for Tial1 KD due to its long protein half-life of 50 h in hepatocytes^46^. While we did not observe differences in proliferation or morphology between *Tial1* KO and parental cells we measured an ≈6-fold increase of INSIG2 in all independent KO clones, thereby confirming our in vivo findings in Tial1 LKO animals (Fig. 4g). To test if the binding of TIAL1 to Insig2 was functional we cloned the 3’UTR of mouse Insig2 into the psiCHECK2 vector, immediately downstream of the open reading frame of the reporter *renilla luciferase*. This construct was then transfected into the Hepa 1–6 *Tial1* KO cells, followed by luciferase activity measurements as a proxy for Insig2 3’UTR regulation. The luciferase activity of the Insig2 3’UTR construct was significantly higher in *Tial1* KO cells compared to the activity in WT cells and empty-control constructs (Fig. 4h). When we overexpressed Tial1 in WT cells using recombinant Ad-Tial1 we measured decreased luciferase activity compared to Ad-Ctrl infected cells (Fig. 4i). This effect was amplified when *Tial1*-depleted Hepa1–6 cells were transfected with the luciferase reporter and Tial1 expression was rescued with Ad-Tial1 and compared to Ad-Ctrl infected cells (Fig. 4i). Lastly, metabolic labeling experiments in primary hepatocytes from wildtype and Tial1 LKO mice revealed increased synthesis of nascent INSIG2 protein in Tial1-deficient cells, while no effect on translation was measured for HuR, a transcript that is not targeted by Tial1 (Fig. 4j). Furthermore, the opposite effect on translational efficiency was found when Tial1 expression in Tial1 LKO cells was rescued by adenoviral mediated Tial1 overexpression (Fig. 4k). Taken together, these results demonstrate a direct interaction between TIAL1 and Insig2 mRNA and suggest that TIAL1 negatively regulates INSIG2 expression post-transcriptionally through binding to the Insig2 3’UTR and enhancing Insig2 translation.

### Modulation of hepatic TIAL1 level regulates triglyceride and cholesterol secretion

**Fig. 5:**
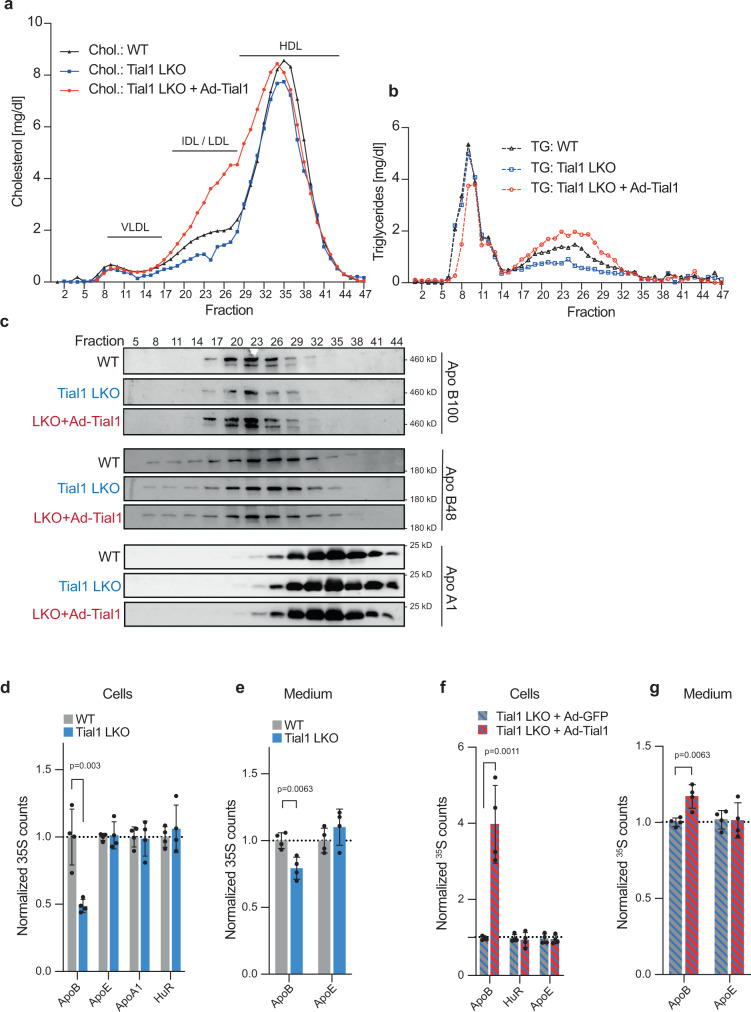
Hepatic ablation and overexpression of TIAL1 regulates cholesterol metabolism. **a** Plasma from mice in (Fig. 3a), was fractionated by FPLC and lipoproteins were quantified through measurements of (**a**) cholesterol and (**b**) triglycerides in each fraction (*n* = 5 per group). **c** Selected fractions from (**a**, **b**) were subjected to western blot analysis of VLDL/IDL/LDL (ApoB48/100) as well as HDL (ApoA1) markers. **d**, **e** ^35^S counts from metabolic labeling and immunoprecipitation of TIAL1 targets and non-targets (controls, HUR, APOE) in primary mouse hepatocytes of WT and Tial1 LKO animals (*n* = 4 per group) (**d**) and their culture media (**e**). ^35^S counts from metabolic labeling and immunoprecipitation of TIAL1 targets and non-targets in primary mouse hepatocytes (*n* = 4 per group) (**f**) and the media (**g**) of Tial1 LKO animals, infected with Ad-GFP or Ad-Tial1. Data are presented as mean values ± SDs. Statistical significance was evaluated by two-tailed Student’s *t*-test. Source data are provided as a Source Data file.

To assess lipoprotein levels and obtain a more complete understanding of the lipoprotein profile in mice with hepatic *Tial1* ablation, we pooled plasma from mutant and wildtype mice and analyzed it by fast phase liquid chromatography (FPLC) and immunoblotting. We found that the changes in plasma cholesterol were also reflected in the lipoprotein profiles, with an overall decrease in plasma cholesterol and triglycerides in Tial1 LKO mice mainly in the APOB containing fractions. Ad-Tial1 injection in Tial1 LKO mice reversed the effect and led to increased cholesterol and triglycerides in the IDL/LDL fractions compared to control animals (Fig. 5a, b).

ViP-CLIP identified the transcript of ApoB as a main target of TIAL1, primarily bound at its CDS and 3’UTR (Supplementary Fig. 5a). Accordingly, we measured decreased circulating APOB100 levels, mainly in IDL/LDL, while APOB48, APOA1 and APOE remained unchanged (Fig. 5c and Supplementary Fig. 5b).

TIAL1 binding sites were localized throughout the 9 kb long 3’UTR of ApoB, which, due to its length, prohibited the use luciferase assays to explore a functional mechanism. We therefore performed pulse chase experiments in primary hepatocytes with L-[35S]- methionine/cysteine, to label newly translated proteins. Following immunoprecipitation of the radiolabeled target proteins, we quantified the translation rate of nascent proteins by scintillation counting. This analysis revealed reduced scintillation counts of APOB in the cells and media derived from Tial1 LKO and control mice, demonstrating that TIAL1 regulates ApoB translation (Fig. 5d, e). In contrast, rescue of TIAL1 expression in Tial1KO hepatocytes using Ad-Tial1 overexpression resulted in increased APOB synthesis (Fig. 5f, g). We also measured mRNA stability of apoB (and Insig2) in Hepa1–6 wildtype and Tial1 KO cells by inhibiting the DNA-dependent RNA polymerase activity with actinomycinD (Supplementary Fig. 5c, d). No significant differences in ApoB and Insig2 mRNA decay were measured, suggesting that Tial1 does not play a major role in mRNA stabilization of these targets.

**Fig. 6:**
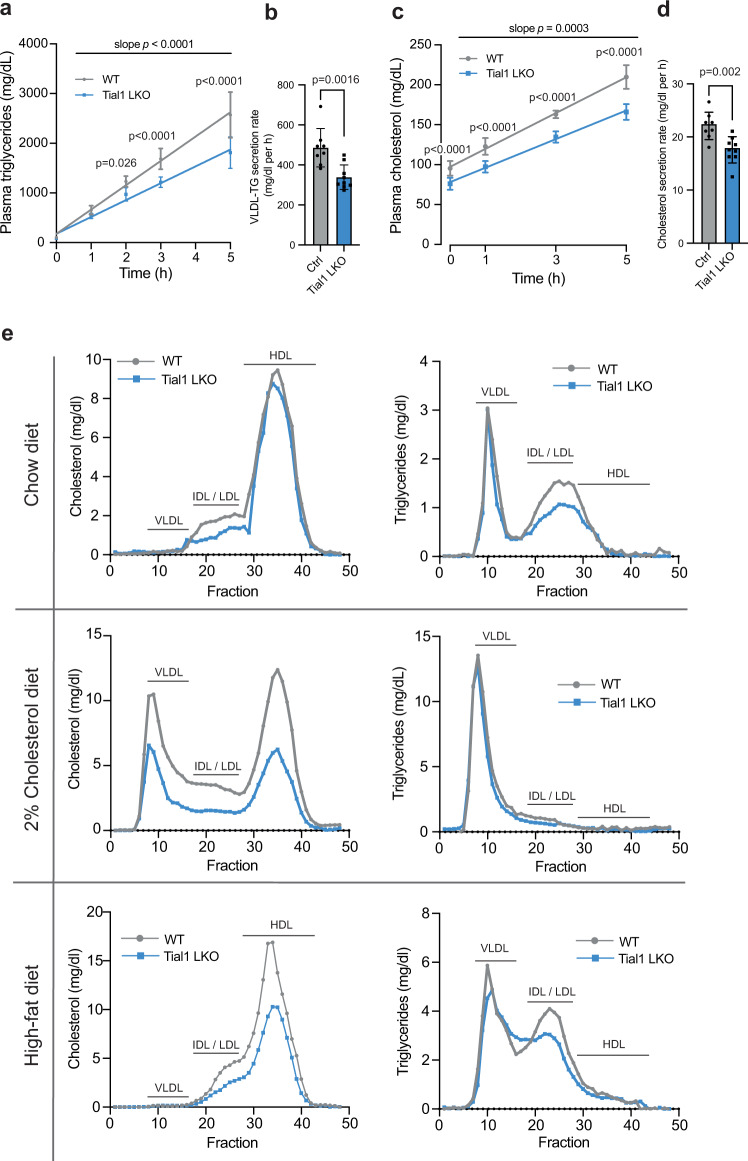
Hepatic inactivation of *Tial1* reduces triglycerides and cholesterol secretion. VLDL-TG and cholesterol secretion assays in 8-week-old (WT; *n* = 8, Tial1-LKO; *n* = 9) mice after 6 h fasting and intravenously injection of 500 mg/kg tyloxapol to block lipases. Plasma was taken at the indicated time-points and triglycerides (TG) (**a**) and total cholesterol (TC) levels (**c**) were determined by enzymatic assay. **b** VLDL-TG and **d** cholesterol secretion rates were calculated from the slopes of increased TG and TC for each mouse. **e** Plasma from mice fed a chow diet, 2% cholesterol diet (*n* = 6) for 3 days *ad libitum* or a high-fat diet for 8 weeks (*n* = 6) was fractionated by FPLC, and lipoproteins were quantified through measurements of cholesterol and triglycerides in each fraction. All data are presented as mean values ± S.D. Statistical significance was evaluated by two-tailed Student’s *t*-test (**b**, **d**) or two-way ANOVA with Holm-Šídâk post hoc analysis (**a**, **c**, **e**). Source data are provided as a Source Data file.

We next hypothesized that the reduction of plasma APOB levels in Tial1 LKO mice may explain the lower plasma VLDL/LDL levels by decreasing the hepatic secretion rate of VLDL, which ultimately results in less VLDL remnants being processed to LDL and accumulation of triglycerides in the liver^47^. Therefore, we determined hepatic VLDL-secretion rates by blocking hydrolysis of triacylglycerides using intravenously injected tyloxapol in fasted mice, thereby hindering the clearance of VLDL particles from plasma. The progressive VLDL accumulation in plasma allows to calculate hepatic triglyceride and lipoprotein secretion rates^48,49^. In accordance with the detected downregulation of APOB100 in Tial1 LKO mice (Fig. 5c), we found that the hepatic triglyceride secretion rate was reduced by 31% compared to control littermate mice (Fig. 6a, b). Consistent with Insig2 regulation, rates of plasma cholesterol in response to tyloxapol injection were also significantly decreased by 21% in Tial1 LKO compared to control mice (Fig. 6c, d).

To investigate if hepatic *Tial1* depletion affects dietary cholesterol utilization and triglyceride secretion under metabolic stress conditions, we challenged mice with high-cholesterol (2%) or high-fat diets. Total cholesterol of control littermates rose significantly to 134% upon cholesterol feeding, while Tial1 LKO prevented the diet induced rise in plasma cholesterol (Supplementary Fig. 6a). Next, we assessed the differences of high-cholesterol diet on the lipoprotein profile. Wildtype mice on a 2% high-cholesterol diet exhibited increased levels of cholesterol in the HDL and chylomicron/VLDL fractions compared to wildtype mice fed a chow diet (0.02% low-cholesterol) (Fig. 6e). Tial1 LKO mice on high-cholesterol diet showed a slight decrease and increase of cholesterol in HDL and chylomicron/VLDL, respectively, while the area under the curve mirrored the overall decreased amount of total cholesterol, as measured in plasma (Fig. 6e). The increase in chylomicron/VLDL was significantly lower in Tial1 LKO compared to control littermate mice, displaying improved protection against the rise of cholesterol upon a cholesterol-rich diet. Similarly, challenging Tial1 LKO and control mice for 8 weeks with a high-fat diet (HFD), displayed lower HDL and IDL/LDL cholesterol in Tial1 LKO compared with control mice (Fig. 6e). Furthermore, triacylglycerol levels were also decreased in VLDL and IDL/LDL fractions. Altogether, these results indicate that hepatic TIAL1 deficiency, in addition to inhibition of an INSIG2/SREBP-mediated reduction in cholesterol and triglyceride synthesis, also decreases the secretion rates of triglycerides and cholesterol via an APOB-mediated mechanism.

## Discussion

RNA-binding proteins (RBPs) are essential in every aspect of RNA existence, including translation, stability, pre-mRNA splicing, 3’UTR processing, and localization^2^. Except for a few reports in brain^50^ and testis^51,52^, current research efforts have focused on studying RBPs using CLIP techniques in cultured cells and nonmammalian model organisms^53–55^. As a result, our knowledge of RBPs and their target composition in mammalian tissues/organs is scarce, which is reflected in a poor molecular understanding of RBP function in physiological and disease states. In addition, RBP-mediated post-transcriptional regulation in the genuine physiological status during metabolic processes in tissues (i.e., amino acid, glucose, lipid, and cholesterol metabolism) has not been addressed. The main reason is that CLIP techniques rely on efficient RBP-RNA crosslinking via UV light, which becomes a limiting factor due to the low penetration depth of short-length light, restricting this approach to 2D cell line cultures.

To address this limitation, we developed viP-CLIP, an in vivo PAR-CLIP protocol, to overcome the constrain of short-length UV light by using the photoactivatable ribonucleoside 4SU. We first performed comprehensive pharmacokinetic studies of 4SU in mice, resulting in a recommended 4SU dose regime over two half-lifes of an average mRNA, which does not lead to toxicities and results in adequate incorporation rates of 4SU in RNA of tissues. 4SU incorporation allowed to increase the crosslinking UV wavelength and improvement of crosslinking efficiency in intact mammalian organs, documented by increased T-to-C conversion in the sequencing reads throughout several organs and RNA classes.

We noticed that depending on the organ, the crosslinking efficiency is not purely defined by its 4SU incorporation rate but also by the abundance of RBPs of interest and their activity. TIAL1 is highly expressed in testis and has been reported to play an indispensable role in germ cell development^24^, and crosslinking is stronger than we would have expected based on the 4SU incorporation rate compared to other organs, such as the heart. We hypothesize that these differences in crosslinking strength among organs could reflect the different functions of RBPs in each organ, their tissue-specific binding partners, their expression level, or the current metabolic environment, which might change the RBP target spectrum. Further, differences in crosslinking can be explained by organ-specific dissimilarities in UV 365 nm penetration depth due to the existence of molecules in the absorption spectra of 4SU. Upon user-specific optimization, our method can in principle be applied to all RBPs^42^, in any tissue and animal models under different metabolic states, opening new opportunities for studying the RBP-RNA interactions in health and disease.

By applying viP-CLIP we describe a previously unrecognized role of TIAL1 in liver as a post-transcriptional regulator of key transcripts in cholesterol metabolism. Cholesterol metabolism is a complex process involving multiple organs and metabolic pathways with many points of regulation that are under genetic and metabolic control.

The liver is the principal site for de novo cholesterol synthesis, dietary cholesterol uptake and excretion, converting cholesterol to bile acids and removing free cholesterol via biliary excretion. Hepatic cholesterol synthesis is regulated by end-product feedback inhibition, demonstrated first in living animals by Schoenheimer in 1933^56^, and is mediated by INSIG proteins, which are required for sterol-mediated inhibition of the processing of sterol-regulatory-element–binding proteins (SREBPs) to their nuclear forms. In this study employing viP-CLIP in livers of mice we identified Insig2 mRNA as a strong TIAL1 target. The Insig2 transcript contained a strong binding site of TIAL1 in a highly conserved region at its 3’UTR, implicated in regulation of its translation^57^. Using reporter assays we show that constructs with the 3’UTR of Insig2 that contains several binding sites had enhanced luciferase activity in Tial1 KO cells, supporting a functional role of TIAL1 in regulating Insig2 transcripts through its 3'UTR. Furthermore, metabolic labeling experiments in primary hepatocytes of Tial1 LKO and following adenoviral overexpression revealed that TIAL1 inhibits protein synthesis of Insig2 transcripts. The negative regulatory effect of TIAL1 on translation corroborates with earlier studies in which selective binding of TIAR to several 3’UTRs of mRNAs potently suppressed their translation^58^. These mRNAs encode translation factors such as eukaryotic initiation factor 4 A (eIF4A), translation initiation factor eIF4E, translation elongation factor eEF1B, and c-Myc, which transcriptionally controls the expression of numerous translation regulatory proteins^57^. However, how precisely TIAL1 represses translation of Insig2 is unclear – future studies will determine if TIAL1 promotes the formation of RNA structures that inhibit ribosome binding or if it traps the ribosome in a complex that hamper effective translation initiation^14^. The elevated levels of INSIG2 in livers and hepatoma cells with ablated *Tial1* are likely responsible for inhibiting the proteolytic processing of SREBP1 and SREBP2, as shown by increased levels of pSREBPs and reduced nSREBPs, and the marked reduction in the levels of mRNAs encoding enzymes required for the synthesis of cholesterol, fatty acids, and triglycerides. Consequently, plasma cholesterol and triglyceride levels were strongly reduced in Tial1 LKO mice compared with controls. This phenotype also mirrors cell lines and transgenic mice overexpressing Insig1 in the liver^32,59–62^, thereby supporting a relevant role of TIAL1 in the negative feedback regulation of INSIG-mediated cholesterol synthesis.

We found that *ApoB* mRNAs is one of the strongest targets of TIAL1 in the liver. APOB availability in the liver is not determined by its transcription^63^, which is relative constant, but by post-transcriptional mechanisms, including autophagy^64,65^, post-translational degradation in the ER^66^, or via post-transcriptional regulation by the RBP VIGILIN (HDLBP)^38^. We have previously demonstrated that the ribosome-associated RBP VIGILIN/HDLBP is a positive regulator of ApoB in the liver by enhancing the translation of a subset of secretory proteins^36^. In this study, we identify TIAL1 as an additional positive regulator of ApoB that directly binds to numerous conserved binding sites in the 3’UTR of ApoB. *Tial1* inactivation leads to a 50% reduction of APOB synthesis, whereas overexpression of TIAL1 markedly increases APOB’s synthesis. TIAL1's effects on plasma APOB levels are less pronounced in high-cholesterol diet conditions, suggesting that it is compensated by other factors. How TIAL1 mediates translational activation of apoB is currently unknown but may be linked to the recruitment of this target mRNAs to translationally active polysomes or TIAL1-mediated exclusion of translational repressors by other RBPs or miRNAs. The impaired APOB expression in Tial1 LKO mice leads to reduced plasma cholesterol levels, decreased VLDL secretion, and accumulation of triacylglycerides in the liver. This phenotype is reminiscent of various models in which the expression of APOB is impaired^67–71^.

RBPs are often multifunctional and interact with a variety of protein partners and display complex localization patterns within cells^44,72^. Recent studies have also shown that they can change their intracellular localization upon metabolic alterations^42,59,73^. Nucleocytoplasmic shuttling of TIAL1, mediated by an RRM3-dependent nuclear export that is independent of CRM1 has been previously reported^44^. The enrichment of TIAL target transcripts in lipid metabolism motivated us to explore if intracellular sterol levels might mediate the subcellular localization of TIAL1 in the liver. We found that cholesterol depletion via the HMGCR inhibitor simvastatin in primary human hepatocytes lead to a strong nuclear localization, while LDL loading had the opposite effect. Both reactions were reversible without alterations of TIAL1 expression level. However, sterol depletion or accumulation did not impact on nucleoplasmic localization of Insig2 and apoB transcripts, indicating that this TIAL1’s subcellular localization can be ruled out as an important regulatory mechanism for translational control of Insig2 and ApoB transcripts.

In the present study, we explored how liver-specific *Tial1* inactivation affects cholesterol by affecting two targets and key regulators of cholesterol metabolism that we identified through an unbiased viP-CLIP analysis of TIAL1 in liver. We cannot exclude that additional TIAL1 targets also contribute to the phenotype or other relevant pathways that are important for maintaining normal liver homeostasis. To get a full understanding of TIAL1 function, or any other RBP, it will be interesting to determine the composition of all RBPs that bind to target mRNAs in physiological and metabolic stress conditions. Furthermore, future studies are warranted that combine genetic RBP ablation, viP-CLIP, and multiomics analysis to dissect the crosstalk of RBPs and their regulatory networks in metabolism and disease.

In concluding, we have developed a robust viP-CLIP protocol, thereby opening the potential to broaden the scope of RBP investigations in vivo by identifying a previously unrecognized function of TIAL1 in cholesterol metabolism. This method may facilitate systematic future efforts to unravel the complex regulatory networks in metabolism that are governed by RNA-RBP interactions.

## Methods

### Experimental animals

Frozen sperm of mice carrying the *Tial1*^*tm1a*^ mutation (C57BL/6 N A^tm1Brd^ Tial1^tm1a(EUCOMM)Wtsi^ / WtsiH) were obtained from the EMMA mouse repository (EMMA ID 09761 and used for in vitro fertilization. Briefly, the *Tial1*^*tm1a*^ KO first allele contains a IRES: LacZ trapping cassette and a floxed promoter-driven neo cassette inserted into the second intron of the *Tial1* gene at position 128056386 of Chromosome 7. *Tial1*^*tm1a*^ mice were initially crossed with Flp-1 transgenic line (Jackson Lab) to remove the FRT-flanked lacZ-neo cassette, converting the “knockout-first” allele to a conditional allele (*Tial1*^*fl/fl*^) (Supplementary Fig. 3a). Successively, hepatocyte-specific knockout animals were obtained by crossing with Albumin-Cre transgenic mice (Jackson Lab) leading to exon 2 deletion and generation of a frameshift mutation. Heterozygous intercrosses resulted in viable and fertile homozygous Tial1 LKO (Tial1^fl/fl^/Alb-Cre). mice maintained on a C57BL/6 N background. Mice were housed in a pathogen-free animal facility at the Institute of Molecular Health Sciences at ETH Zurich, in a temperature-controlled room (22 °C), with humidity at 55% and on a 12 h light−dark cycle (lights on from 6:00 to 18:00). Mice were fed standard laboratory chow, a high-fat diet (fat, carbohydrate, protein content was 45, 35, and 20 kcal%, respectively) (Research Diets, D12451), a chow diet supplemented with 2% cholesterol, and water *ad libitum*. All ethical regulations were complied with, and all animal experiments were approved by the Kantonale Veterinäramt Zürich. Unless otherwise indicated in the figures and figure legends, all experiments were performed in randomly chosen age-matched male mice using littermates as controls. All WT control animals were littermates (*Tial1*^*fl/fl*^).

### Adenoviral infections

Mouse Myc-DKK tagged cDNAs of Tial1 (NM_00009383) were provided by Origene and cloned into pVQAd CMV K-NpA (Viraquest) using the restriction sites KpnI and PmeI (Ad-Tial1). All viruses expressed GFP from a separate adenoviral locus than the transgene. Ad-GFP control was based on the same vector backbone (including GFP). Cells were infected with MOIs indicated in figures and legends for 6 h and then maintained in culture until the indicated time points. Adenoviral infection of mice was performed by a single tail vein injection of 3 × 10^9^ plaque-forming units in a final volume of 0.2 ml diluted in PBS.

### Mouse primary hepatocytes isolation

Primary hepatocytes were isolated from male mice 8 weeks of age via collagenase perfusion, as previously described in ref. ^38^, with minor modifications. Mice were anaesthetized by intraperitoneal injection of pentobarbital (Esconarkon US vet). The liver was perfused by cannulation of the inferior vena cava with the hepatic portal vein as a drain. The liver was perfused with pre-warmed Hank’s Balanced Salt Solution (HBSS, Life Technologies) containing 0.5 mM EGTA followed by pre-warmed digestion medium (DMEM 1 g/l glucose (Life Technologies), 1% Penicillin–Streptomycin (Life Technologies), 15 mM HEPES (Life Technologies), 30 μg ml^−1^ Liberase Research Grade medium Thermolysin concentration (Roche)) each for five minutes with a flow rate of 5 ml/min. The liver was surgically removed, hepatocytes released into 10 ml digestion media by shaking and supplemented with 15 ml ice-cold low glucose media (DMEM 1 g/l glucose (Life Technologies), 1% Penicillin–Streptomycin (Life Technologies), 10% heat-inactivated fetal bovine serum (Sigma-Aldrich), 1% GlutaMax (Life Technologies)) and filtered through a 100 μm Cell Strainer (BD). The suspension was then washed three times with 25 ml of ice-cold low glucose media and centrifuged at 50 x *g* and 4 °C for 2 min. Hepatocytes were counted and plated at 0.4 ×10^6^ cells surface-treated 6-well plates (BD Primaria) in low glucose media. Three hours after plating, cells were washed once with PBS and medium was changed to Williams E medium (or methionine-free DMEM for cell extracts used for in vitro translation; Life Technologies) supplemented with 1% penicillin–streptomycin (Life Technologies), 1% GlutaMax (Life Technologies). All cells were incubated at 37 °C in a humidified atmosphere containing 5% CO_2_.

### Isolation of Primary Human Hepatocytes

Primary human hepatocytes were obtained from CytesBiotech. The cells used in our experiments were obtained from a male, 62-year-old, Caucasian, BMI 23, nonsmoker, nondrinker, and nondrug user. Cells were tested and found to be free of the following pathogens: human immunodeficiency virus I/II, human T-cell lymphotrophic virus I/II, HBsAG, HBcAB, hepatitis C virus and cytomegalovirus. 8×10^6^ cells were thawed in 45 ml Cryopreserved Hepatocyte Recovery Medium (Gibco, CHRM, GB700), and recovered by centrifugation at 100 g for 10 min. The hepatocytes were seeded in a 24-well plate with a density of 0.3 × 10^6^ cells/well in 0.5 ml plating media (William’s Medium E, A1217601, supplemented with hepatocyte plating supplement pack CM3000, Gibco, ThermoFisher). After a 6 h incubation, the cells were washed with PBS and cultured in incubation media (William’s Medium E, A1217601, supplemented with hepatocyte maintenance supplement pack, CM4000, Gibco, ThermoFisher). Cells had a post-thaw viability of 92% and were plated on collagenized plates and coverslips. All cells were incubated at 37 °C in a humidified atmosphere containing 5% CO_2_.

### Blood plasma collection and measurements

For measuring blood plasma insulin, ALT (Bioassay), AST (Bioassay), triglyceride, cholesterol, and NEFA levels, blood was collected from the submandibular vein in nonheparinized capillary tubes. EDTA was added to a final concentration of 5 mM as an anticoagulant. Plasma was then separated by centrifugation at 8,000 g for 4 min. Measurements were performed using commercial kits. Plasma cholesterol (Infinity^TM^ cholesterol kit), triglycerides (Infinity^TM^ triglyceride kit), NEFA (Wako) and bile acids (Dyazyme) were measured by colorimetric assays, according to the manufacturer’s instructions. Blood glucose was measured using a Contour glucometer (Bayer).

### In vivo *VLDL* secretion assay

Mice were fasted for 6 h and injected intravenously with the lipase inhibitor tyloxapol (500 mg/kg; Sigma) prior to blood collection at the indicated time points in the figures and figure legends after injection. The collected blood samples were used for TG and TC measurements and the VLDL-TG and -TC production rate was calculated from the slope of the plasma TG and TC versus time curve.

### Metabolic labeling

Primary hepatocytes isolated from WT and Tial1 LKO mice were cultured overnight, washed twice with PBS and incubated for 2 h in methionine/cysteine free medium. Cells were then pulsed for 2 h with 150 μCi of [^35^S]-methionine/cysteine (Expre^35^S^35^S; PerkinElmer) per well and chased for 6 h in full hepatocytes medium after washing with PBS. Metabolically labeled cells were harvested and lysed in 1 × NP40 lysis buffer (50 mM HEPES, pH 7.5, 150 mM KCl, 0.5 mM EDTA, 1 mM NaF, 0.5% (v/v) NP40, 50 μM DTT, cOmplete^TM^ EDTA-free protease inhibitor cocktail (Roche)) and incubated on ice for 10 min. Lysates and cell culture media were cleared by centrifugation at 13,000 g for 10 min. The supernatant was collected and used for immunoprecipitation. TIAL1 targets and nontargets were immunoprecipitated with target-specific antibodies conjugated to protein G Dynabeads overnight at 4 °C. Ten microliters of protein G magnetic particles were used per ml cell lysate or cell culture media. Magnetic beads were collected on a magnetic rack and washed with 1 × NP40 lysis buffer three times before quantification using the scintillation counter.

### RNA isolation, quantification, and sequencing

RNA was extracted using Trizol (Life Technologies) according to the manufacturer’s instructions, except for a 30 min isopropanol precipitation at −20 °C. RNA integrity was analyzed on an Agilent 2100 Bioanalyzer for all samples that were sequenced. RNA was subjected to DNase I treatment with the DNA-free kit (Invitrogen), when necessary. RNA was reverse transcribed using the High-Capacity cDNA Reverse Transcription Kit (Applied Biosystems). Quantitative PCR was performed in an LC480 II Lightcycler (Roche) and using gene-specific primers and Sybr Fast 2x Universal Master mix (Kapa). Results were normalized to 36B4 or Actin mRNA levels. Primers used for qPCR are shown in Supplementary Table 1. The samples used for RNA sequencing were further purified using RNeasy columns (QIAGEN, #74104). RNA integrity was analyzed using Agilent 2100 Bioanalyzer for all samples. For RNA-sequencing standard methods were used, the cDNA library preparation was constructed using TruSeq mRNA library prep kit from Illumina and the sequencing was performed at the Functional Genomics Center Zurich (FGCZ) on a Novaseq 6000 platform. Results from 3 individual livers per genotype (WT and Tial1 LKO) were analyzed.

### Liver triglyceride and cholesterol content

Lipids from 50 mg liver were extracted with 1 ml hexane:isopropanol (3:2) by homogenizing tissues using the TissueLyser II (Qiagen). Lysates were centrifuged at 20,000 g for 3 min and the supernatant was transferred to a fresh tube. The pellet was re-extracted with 0.5 ml hexane:isopropanol, spun again and the supernatants were combined. A volume of 0.5 ml of 0.5 M Na_2_SO_4_ solution was added and the tubes were mixed. The samples were centrifuged for 3 min at full speed and the upper organic phase was transferred to a fresh tube, avoiding contamination with the aqueous phase. The samples were spun again, and the upper phase was transferred to a fresh tube and evaporated overnight under the fume hood. Lipids were dissolved in 1 ml of Triton X-100:methanol:butanol (1:1:3) mixture and TC and TG level were quantified.

### Plasma fractionation

Lipoproteins from pooled plasma were separated by FPLC by loading 400 μL pooled plasma into a sample loop. After loading of the sample, fractions of 0.5 mL were separated by using two Superose-6 FPLC columns in series (HR10/30) in FPLC buffer (0.15 M NaCl, 0.01 M Na_2_HPO_4_, 0.1 mM EDTA, pH 7.5) at 0.4 ml/min. Columns were calibrated using high and low molecular weight standards (GE Healthcare).

### Western blot analysis

Cells and tissues were homogenized (using the Tissue Lyser II, Qiagen) with 3 volumes of RIPA lysis buffer (50 mM Tris-HCl pH 8, 150 mM NaCl, 1% NP40, 0.5% sodium deoxycholate, 0.1% SDS and 1 tablet cOmplete^TM^ EDTA-free protease inhibitor cocktail (Roche)), incubated for 30 min on ice and centrifuged for 15 min at 16,000 × *g* and 4 °C. Protein concentrations were determined using the BCA assay (Sigma-Aldrich). Equal protein amounts were boiled in Laemmli buffer (1.7% SDS, 5% glycerol, 0.002% bromophenol blue, 60 mM Tris-HCl, pH 6.8, 100 mM DTT) for 5 min at 95 °C, separated by SDS–PAGE and transferred onto nitrocellulose membranes by electroblotting with the semi-dry Trans-Blot Turbo Transfer system (BioRad). The membranes were blocked for 1 h with 5% nonfat dry milk TBS-0.1% Tween (Sigma-Aldrich), incubated with the primary antibodies overnight at 4 °C, followed by three washes in TBS-0.1% Tween and incubation with a horseradish peroxidase-conjugated secondary antibodies (Calbiochem) for 2–3 h. Blots were then developed by chemiluminescent detection with a Fujifilm analyzer (LAS-4000) and signals quantified using ImageJ.

### Nuclear and Cytoplasmic Extracts

Cells were washed twice with cold phosphate-buffered saline, scraped on ice, pelleted, and resuspended in hypotonic lysis buffer (10 mM HEPES (pH 7.9), 1.5 mM MgCl_2_, 10 mM KCl, 1 mM DTT, cOmplete^TM^ protease inhibitor cocktail (Roche), 0.1% NP40). Lysates were incubated on ice for 1 min, vortexed briefly and centrifuged at 10,000 × *g* for 30 s at 4 °C. The supernatants (cytoplasmic extracts) were collected, and the nuclear pellets were resuspended in RIPA buffer (50 mM Tris-HCl, pH 8, 150 mM NaCl, 1% NP40, 0.5% sodium deoxycholate, 0.1% SDS and 1 tablet cOmplete^TM^ EDTA-free protease inhibitor cocktail (Roche)). Protein concentrations were determined by the BCA assay (Sigma).

### Single-molecule fluorescent in situ hybridization

For single-molecule fluorescent in situ hybridization (smFISH), 1 ×10^5^ primary human hepatocytes were seeded on precision coverslips (Marienfeld, 0117580), and cultured for 6 h until attached and treated with Simvastatin (Merck S6196) at the indicated concentration for 16 h. Cells were fixed in 4% paraformaldehyde for 5 min, washed and smFISH was performed as described previously^74^. Probes targeting the ApoB open reading frame (Supplementary Table 2) were enzymatically labeled with Atto-565 using terminal deoxynucleotidyl transferase^75^. Imaging was performed using a Zeiss AxioObserver7 inverted microscope equipped with a Yokogawa CSU W1-T2 spinning disk confocal scanning unit, a Plan Apochromat 100 × 1.4 NA oil objective, and an sCMOS camera. Image acquisition was performed using VisiView software, version 5.0.0.16. Z-stacks were acquired in steps of 0.2 μm over a total z-depth of 5.2 μm. Exposure times were 800 ms and 50 ms for 561 and 405 channels, respectively.

### CRISPR-Cas9 genome engineering for generating knockout Hepa 1–6 cell lines

Cas9-mediated genome editing of Hepa 1–6 cells was performed as previously described in ref. ^76^. The oligodeoxynucleotides encoding sgRNAs (forward sequence: CACCGAAATCAGCATTTGCCCCCTT, reverse sequence: AAACAAGGGGGCAAATGCTGATTTC) for targeting the coding region of the gene were annealed and ligated into pSpCas9(BB)-2A-GFP (Addgene, PX458, Plasmid #48138) linearized with BbsI, and plasmids were sequenced after cloning and transformation. To generate gene knockout Hepa 1–6 cells, we transfected 100’000 cells with the corresponding guide sequence containing pSpCas9(BB)-2A-GFP plasmid. Overall, 24 h after transfection, GFP-positive cells were sorted clonally by FACS into 96-well plates and cultivated until colonies were obtained. Clonal cell lines were analyzed by immunoblot for protein depletion.

### Cell culture conditions Hepa 1–6 and HEK 293

Hepa 1–6 cells (ATCC, #CRL-1830) and HEK 293 T cells (Dharmacon, #HCL4517) were cultured in high-glucose DMEM (Thermo Fisher Scientific, 11965118) supplemented with 10% v/v FBS, 100 U/ml penicillin, 100 μg/ml streptomycin, 2 mM L-glutamine. All cells were incubated at 37 °C in a humidified atmosphere containing 5% CO_2_.

### Antibodies

The following antibodies were used in immunoblotting: mouse anti-γ-tubulin (1:10,000) (Sigma-Aldrich, #T6557), rabbit anti-Gapdh (1:500) (Santa Cruz, #2118 S), rabbit anti-HuR (1:500) (Santa Cruz, #sc-20694), rabbit anti-Histone H3 (1:5,000) (Cell Signaling, #4499 S), rabbit anti-apoB (1:2,000) (Meridian, #K23300R), rabbit anti-apoE (1:2,000) (Meridian, #K23100R), rabbit anti-apoA-I (1:10,000) (Meridian, #K23500R), mouse anti-Tial1 (1:1000) (BD Biosciences, #610352), mouse anti-Myc (1:1000) (Millipore, clone 4A6, #05-724), rabbit anti-ßActin (1:5000) (Cell Signaling, #4970 S), rabbit anti-Insig2 (1:250) (ProteinTech, #24766), rabbit anti-Srebp2 (1:500) (ThermoScientific, #PA5-88943), rabbit mouse-Srebp1 (1:500) (ThermoScientific, #MA516124), rabbit anti-Insig1 (1:500) (Abcam, #ab70784), rabbit anti-HMGCR (1:500) (ABclonal, #A19063), rabbit anti-Scap (1:500) (ThermoScientific, #PA5-28982), rabbit anti-HSP90 (1:1000) (Cell Signaling, #4874 S). The following secondary HRP conjugated Antibodies were used in immunoblotting: Goat anti-mouse IgG-HRP (1:10000) (Calbiochem, #401253), Goat anti-rabbit IgG-HRP (1:10000) (Calbiochem, #401393).

### Measurement of 4SU incorporation levels into total RNA

Total RNA was isolated from the tissues of mice injected with 4SU or from cell lines grown in medium supplemented with 100 μM 4SU 16 h prior to harvest. As a control, cells grown without 4SU, and mice injected with PBS were used. The RNA was isolated with Trizol reagent (Sigma) following the manufacturer’s instructions. To prevent a possible oxidization of 4SU during RNA isolation and analysis 0.1 mM DTT was added to the wash buffers and subsequent enzymatic steps. The RNA was digested and dephosphorylated to single nucleotides as described before^77^. Briefly, 40 μg of purified total RNA in 30 µL were incubated for 16 h at 37 °C with 0.4 U bacterial alkaline phosphatase (Worthington Biochemical) and 0.09 U snake venom phosphodiesterase (Worthington Biochemical). As a reference standard, a synthetic 4SU-labeled RNA oligonucleotide (sequence: CGUACGCGGAAUACUUCGA(4SU)U) was used and subject to the same enzymatic digestion. The single ribonucleoside- containing reaction mixtures were separated via HPLC on a Supelco Discovery C18 (bonded phase silica 5 μM particle, 250 ×4.6 mm) reverse phase column (Sigma). The separation was performed with an isocratic gradient: 0% B for 15 min, 0 to 10 % B for 20 min, 10 to 100% B for 30 min, mobile phase (A) 3% acetonitrile in 0.1 M TEAA (92:5:3 deionized water: 2 M TEAA: acetonitrile) and mobile phase (B) 90% acetonitrile in water. The HPLC column was cleaned between the runs with 100% (B). The base composition is calculated from the integrated areas of the oligonucleotides.

### In vivo PAR-CLIP

HPLC purified ( ≥ 98%) 4-thiouridine (4SU) was purchased from Tocris (Cat No. 7005), protected against light, and dissolved fresh before use. For in vivo PAR-CLIP mice were injected intraperitoneally with 780 mg/kg 4-thiouridine (4SU) every 2.5 h over a time-course of 12.5 h. Mice were sacrificed 2.5 h after the last injection, the organ or tissue of interest surgically removed, and flash frozen in liquid nitrogen. The tissue was grinded in liquid nitrogen to a fine powder and irradiated on a customized liquid nitrogen cool-pad four times with 0.300 J × cm^−2^ of 365 nm UV light, with mixing of the powder in between the irradiations. The pilot scale viP-CLIPs (Fig. 1) were performed with approximately 250 mg of tissue powder lysed in 1 mL NP40 lysis (50 mM HEPES-K, pH 7.5, 150 mM KCl, 2 mM EDTA. 1 mM NaF, 0.2% (v/v) NP40, 0.5 mM DTT, cOmplete^TM^ EDTA-free protease inhibitor cocktail, PhosSTOP phosphatase inhibitor cocktail). The large scale viP-CLIP of liver (Fig. 2) was done with approximately 2 g of irradiated liver powder in 10 mL NP40 lysis (50 mM HEPES-KOH, pH 7.5, 150 mM KCl, 2 mM EDTA. 1 mM NaF, 0.06% (v/v) NP40, 0.5 mM DTT, cOmplete^TM^ EDTA-free protease inhibitor cocktail, PhosSTOP phosphatase inhibitor cocktail). The lysate was homogenized with 10 strokes on ice in a glass douncer, sonicated for 5 cycles at”low” 30 s on/off, cleared by centrifugation at 13,000 g at 4 °C for 20 min and filtered through a 0.45 μm membrane filter.

Myc-tagged Tial1 from livers in mice injected 6 days prior to 4SU injections with Ad-Tial1, was immunoprecipitated with anti-myc antibodies conjugated to 250 μL of Protein G magnetic beads per ml tissue lysate over night at 4 °C. Endogenous Tial1 was immunoprecipitated with anti-Tial1 antibodies conjugated to 350 μL of Protein G magnetic beads per mL tissue lysate over night at 4 °C. Beads were washed 3 times in IP-wash buffer (50 mM HEPES-K, pH 7.5, 200 mM KCl, 0.05% (v/v) NP40) and resuspended in one bead volume IP-wash buffer and treated with RNase T1 at a final concentration of 0.13 U/μl and incubated at 37 °C for 5 min. Beads were washed three times in ice cold high salt buffer (50 mM HEPES-KOH, pH 7.5, 500 mM KCl, 0.05% (v/v) NP40) and resuspended in one bead volume of dephosphorylation buffer (50 mM Tris-HCl, pH 7.9, 100 mM NaCl, 10 mM MgCl_2_, 1 mM DTT). Calf intestinal phosphatase was added to a final concentration of 0.5 U/μl, incubated for 30 min at 37 °C and washed twice in phosphatase wash buffer (50 mM Tris-HCl, pH 7.5, 20 mM EGTA, 0.5% (v/v) NP40). The crosslinked RNA was labeled with γ-^32^P-ATP to a final concentration of 0.5 μCi/μl and T4 PNK to 1 U/μl in the original bead volume for 30 min at 37 °C. Nonradioactive ATP was added to obtain a final concentration of 100 μM and incubated for another 15 min at 37 °C. The radiolabeled band corresponding to the RBP-RNA complex was separated by SDS–PAGE and transferred to a nitrocellulose membrane, detected, and cut out. The membrane pieces were incubated in proteinase K buffer (50 mM Tris-HCl, pH 7.5, 75 mM NaCl, 5 mM EDTA, 1% (w/v) SDS) containing 1 mg/ml proteinase K and incubated for 30 min at 50 °C. The RNA was recovered by acidic phenol/chloroform extraction and ethanol precipitation. The cDNA library was prepared by ligation of pre-adenylated 3′ adapter and 5′ adaptor RNA before reverse transcription and PCR amplification. The PCR product corresponding to the insert ligated to both adapters were cut out from a 2.5% agarose gel and purified using Qiaquick gel purification kit. The amplified and purified cDNA was submitted for sequencing at the Rockefeller University Genomics Center. The data was analyzed using the PAR-CLIP suite^5^ and PARalyzer^78,79^. Mapping statistics and the result from both replicates are listed in Source data. A detailed step-by-step protocol is available in *Supplementary Methods*.

### Motif analysis

Motif analysis was carried out calculating 4-mer to 8-mer enrichments using MEME suite, MEME (http://meme-suite.org/tools/meme) to define the motif of the top 1000 in 3′UTR and introns as defined by PARalyzer, which had at least 10 reads.

### Luciferase assays

The WT Insig2 3′UTR was PCR-amplified from genomic DNA of WT mice (fw primer: ACGCCTCGAGAGAGGGCAGACGTCTTATCT, rv primer: ATAGTTAGCGGCCGCTGGTGATAATTACATATTTAAT) and cloned into the PsiCHECK2 (Promega) using the restriction sites XhoI and NotI (NEB). Cells were transfected using Lipofectamin2000 according to the manufacturer’s instructions. Cells were harvested and assayed using the Dual-Luciferase Reporter Assay System (Promega). The PsiCHECK2 vector contains a second reporter, firefly luciferase, that was used to normalize the results of *Renilla* luciferase expression upstream of the inserted 3’UTR.

Liver RNA Extraction, Sequencing, RT-PCR Validations, and Real-Time qPCR

Total RNA was prepared from liver pieces of central lobe through homogenization in Trizol reagent (Sigma) using a Tissue Lyser II (QIAGEN). To each sample 0.2 volumes of chloroform were added, centrifuged for 15 min at 12,000 x g at 4 °C and finally the upper aqueous phase was precipitated with isopropanol for 30 min at −20 °C. After resuspension in water, RNA samples were digested with 10 units of DNase I for 15 min at 37 °C following a phenol/chloroform extraction and ethanol precipitation. The samples used for RNA sequencing were further purified using RNeasy columns (QIAGEN, #74104). RNA integrity was analyzed using Agilent 2100 Bioanalyzer for all samples. For RNA-sequencing standard methods were used, the cDNA library preparation was constructed using TruSeq mRNA library prep kit from Illumina and the sequencing was performed at the Functional Genomics Center Zurich (FGCZ) on a HiSeq 4000 platform.

### RNAseq

Isolated RNA concentration and quality were assessed with a Qubit (1.0) Fluorometer (Life Technologies) and a Bioanalyzer 2100 (Agilent), respectively. Samples with a 260/280 nm ratio between 1.8 and 2.1 and a 28 S/18 S ratio within 1.5 and 2.0 were further processed for sequencing. TruSeq RNA Sample Prep Kit v2 (Illumina) was used for cDNA library preparation and quality and quantity of the enriched libraries were validated using Qubit (1.0) Fluorometer and the Caliper GX LabChip GX (Caliper Life Sciences). Libraries were normalized to 10 nM and sequenced on the Illumina HiSeq 4000 at the Functional Genomics Center Zurich. Pathway enrichment analysis and upstream regulator analysis were performed using Ingenuity (Qiagen).

### Statistics and reproducibility

Numerical values are represented as mean values ± SD unless stated otherwise. No statistical method was used to predetermine sample size, but sample size was based on preliminary data and previous publications as well as observed effect sizes. Outliers that were two standard deviations outside of the mean were excluded from all analyses. Animals were sex- and age-matched. Investigators were blinded to group allocation during data collection, sample collection/processing, and analysis. Except for animal experiments, all experiments were repeated once with similar results. We assessed data for normal distribution and similar variance between groups using GraphPad Prism 9.0 if applicable. If not mentioned otherwise in the figure legend, statistical significance was determined by unpaired two-tailed t-test, one-way ANOVA (when comparing ≥3 groups) or two-way ANOVA (for repeated measurements and time courses) with relevant post hoc tests (Holm-Sidak for=3 groups and Tukey’s for repeated time measurements and time courses). GraphPad Prism 9.0 software was used for statistical analysis of all data sets.

### Reporting summary

Further information on research design is available in the Nature Portfolio Reporting Summary linked to this article.

## Supplementary information

Supplementary Information

Reporting Summary

## Data Availability

The data supporting the findings of this study are available within the article and its Supplementary Information Files. The differential expression and PAR-CLIP data reported in this study are available at NCBI SRA with the accession number PRJNA869588. Source data are provided with this paper.

## Source data

Source Data
