# Supplementary material for: In vivo PAR-CLIP (viP-CLIP) of liver TIAL1 unveils targets regulating cholesterol synthesis and secretion
Source: Nat Commun. 2023 Jun 9;14:3386. doi: 10.1038/s41467-023-39135-8 (PMC10256721; doi:10.1038/s41467-023-39135-8)
Supplement: Supplementary file 1 — Supplementary Information [file 41467_2023_39135_MOESM1_ESM.pdf]

SUPPLEMENTARY FIGURES

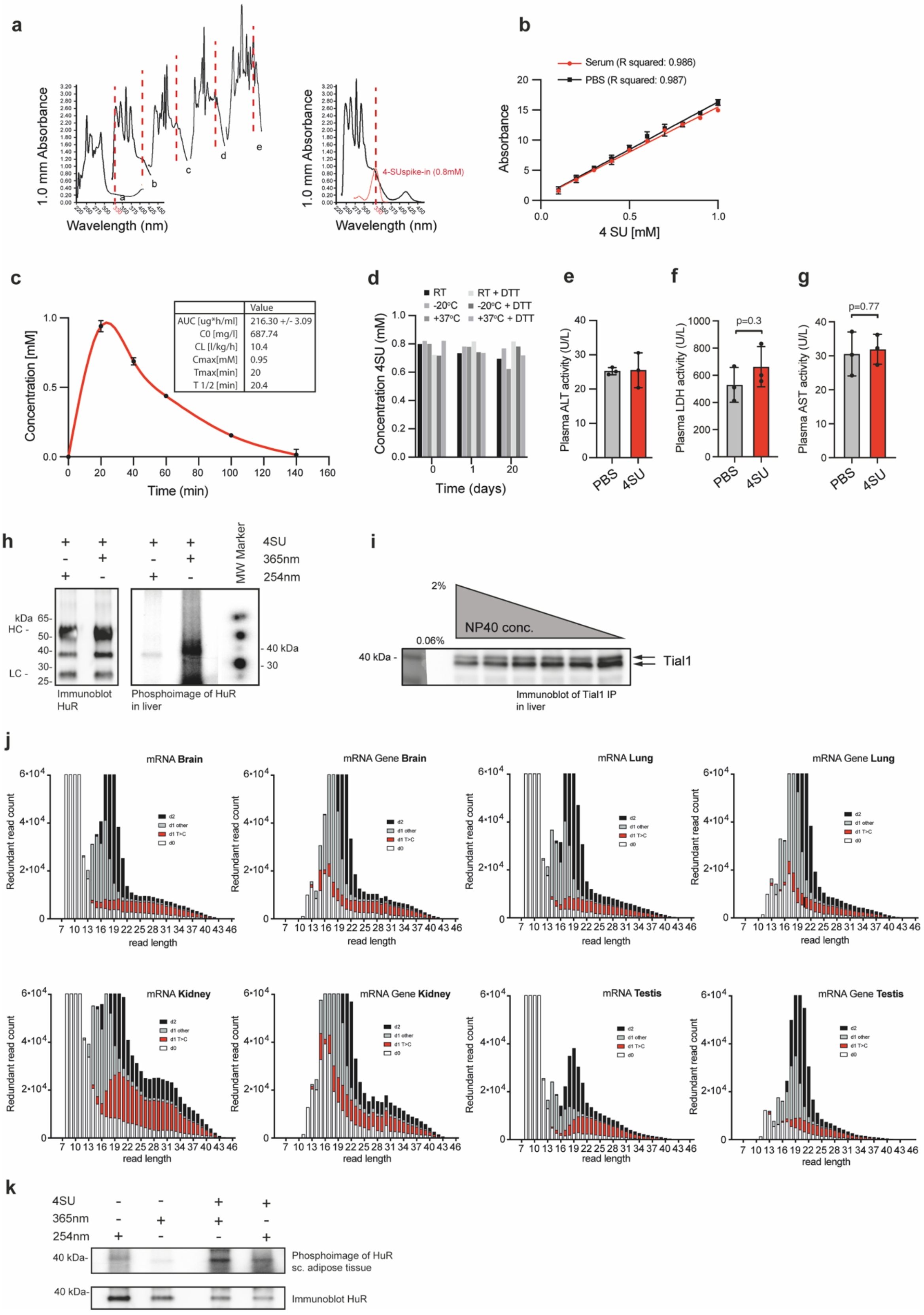

Supplementary Figure 1: 4SU determination, pharmacokinetics, T-to-C conversion in tissues sequencing reads. ***(continued on next page)***

Supplementary Figure 1: 4SU determination, pharmacokinetics, T-to-C conversion in tissues sequencing reads.

(**a**) Left: Spectrograms of serum spiked with 4SU. The dashed red line marks the wavelength of 330 nm. (a) blank mouse serum, (b) bank mouse serum spiked with 0.4 mM 4SU, (c) blank mouse serum spiked with 0.8 mM 4SU, (d) blank mouse serum spiked with 1.2 mM 4SU, (e) blank mouse serum spiked with 2 mM 4SU. Right: UV spectrum of blank mouse serum spiked with 0.8 mM 4SU (black graph) overlaid with a spectrum of water spiked with 0.8 mM 4SU (red graph).

(**b**) Linearity range of 4SU measurements in PBS and serum, spiked with indicated concentrations of 4SU (n=3 animals/independent experiments).

(**c**) Pharmacokinetic study of 4SU serum concentration after a single dose i.p. injection in mice (n=5 independent animals). Corresponding pharmacokinetic parameters are shown in the table included in the graphic.

(**d**) Time and temperature effect on serum spiked with 0.8 mM 4SU. 4SU concentration in serum samples of injected mice spiked at 0.8 mM 4SU. The dashed graphs are samples spiked with 4SU and supplemented with 2 mM DTT. The samples were stored in at RT, -20°C, and +37°C for 0, 1 and 20 days.

(**e–g**) Plasma alanine transaminase (ALT) (e), lactate dehydrogenase (LDH) (f), and aspartate transaminase (AST) (g) activity levels from mice (n=3) receiving six i.p. injections of 4SU or PBS every 2.5 h or 12.5 h over a total period of 15 h, as shown in Fig. 1a.

(**h**) Autoradiograph and anti-HuR immunoblot of immunoprecipitates of HuR liver viP-CLIP on nitrocellulose after 5'end radiolabeling and SDS-PAGE separation. HC: heavy chain, LC: light chain.

(**i**) Immunoblot of TIAL1 immunoprecipitates from liver tissue conducted in decreasing amounts of NP40 in the lysis buffer.

(**j**) Tial1 viP-CLIP in brain, lung, kidney, and testis displaying sequencing read composition of mRNA and mRNA precursor categories. Reads were assigned as d0 (white), d1 T-to-C (red), d1 other than T-to-C, (light grey), and d2 (black).

(**k**) Autoradiograph and anti-HuR immunoblot of immunoprecipitates of HuR subcutaneous adipose tissue viP-CLIP on nitrocellulose after 5'end radiolabeling and SDS-PAGE separation.

At least three independent replicates were performed obtaining similar results (for e–g, h–i, k). All data are represented as mean values ± S.D. Statistical significance was evaluated by two-tailed Student's *t*-test (e–g). Source data are provided as a Source Data file.

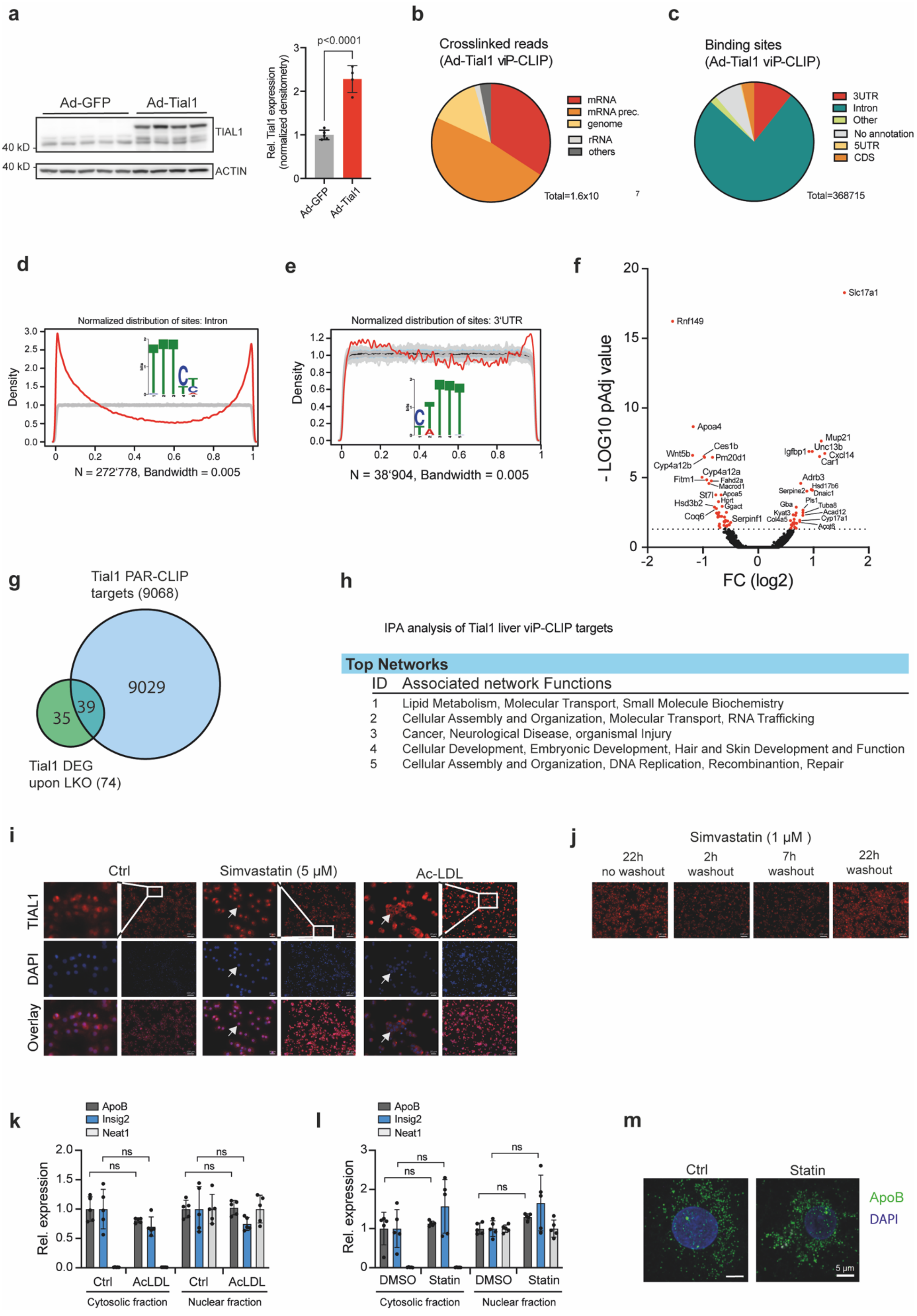

Supplementary Figure 2: Overexpressed tagged TIAL1 crosslinks and binds to target mRNAs involved in lipid metabolism. ***(continued on next page)***

Supplementary Figure 2: Overexpressed tagged TIAL1 crosslinks and binds to target mRNAs involved in lipid metabolism.

(**a**) Western blot and quantification of TIAL1 expression in livers injected with control Ad-GFP (n=5) or Ad-Tial1 (n=4).

(**b**) Crosslinked Ad-Tial1 liver viP-CLIP reads harboring characteristic T-to-C conversions primarily map to the precursor mRNA and mRNA categories.

(**c**) PARalyzer-based binding sites distribution in various RNA-species primarily indicates intronic and 3'UTR binding.

(**d**) Normalized density distribution of Ad-Tial1 liver viP-CLIP binding sites (red line) over 3'UTRs compared to a randomized background (gray line). TIAL1 binding sites accumulate close to the poly adenylation and cleave sites. Sequence logo for the RNA recognition element of TIAL1 top 1000 3'UTR sequence read clusters is indicated in the graph.

(**e**) Normalized density distribution of Ad-Tial1 liver viP-CLIP binding sites (red line) over introns compared to a randomized background (gray line). An enrichment of TIAL1 binding at 5′ and 3′ splice sites is observed. Sequence logo for the RNA recognition element of TIAL1 top 1000 intronic sequence read clusters is indicated in the graph.

(**f**) Volcano plot of the -log10 (adjusted *p* value) against the log fold-change (FC(log2)) of the differentially expressed genes upon *Tial1* liver knockout (retrieved from Table S5). Horizontal dotted line indicates adjusted *p* value = 0.05 (red dots indicate significant regulation). *P* values were determined by a two-tailed Wald test with Benjamini–Hochberg adjustment.

(**g**) Overlap of TIAL1 liver viP-CLIP targets with differentially expressed genes of liver Tial1 LKO mice (retrieved from Table S5).

(**h**) Ingenuity Pathway Analysis (IPA) of the top 500 TIAL1 targets in liver identified by viP-CLIP analysis.(**i**) Additional representative images of TIAL1 immunostainings in primary human hepatocytes treated with DMSO (vehicle control), AcLDL (120 µM), or simvastatin (5 µM) for 24 h.

(**j**) Comparison of cells treated for 46 h with statin (left) and 24 h with statin and then washed and cultured for additional 2 h, 7 h and 22 h without statin.

(**k, l**) Quantitative PCR of apoB, Insig2 and Neat1 (nuclear control transcript) RNAs from cytosolic and nuclear fractions of primary human hepatocytes that were treated with AcLDL (50 µg/ml) (k), simvastatin (5 µM) (l) and respective controls for 16 h. n=5 biologically independent experiments.

(**m**) Single molecule fluorescent in situ hybridization (FISH) analysis of representative human primary hepatocyte cultured with simvastatin (5 µM) or control media for 16 hrs prior to labeled with apoB probes. DAPI was used as a nuclear counterstain. Size bar: 5 µM. Two independent replicates were performed (for i–l, m).

Data in a,k,l are represented as mean values ± S.D. Statistical significance was evaluated by two-tailed Student's *t*-test. Source data are provided as a Source Data file.

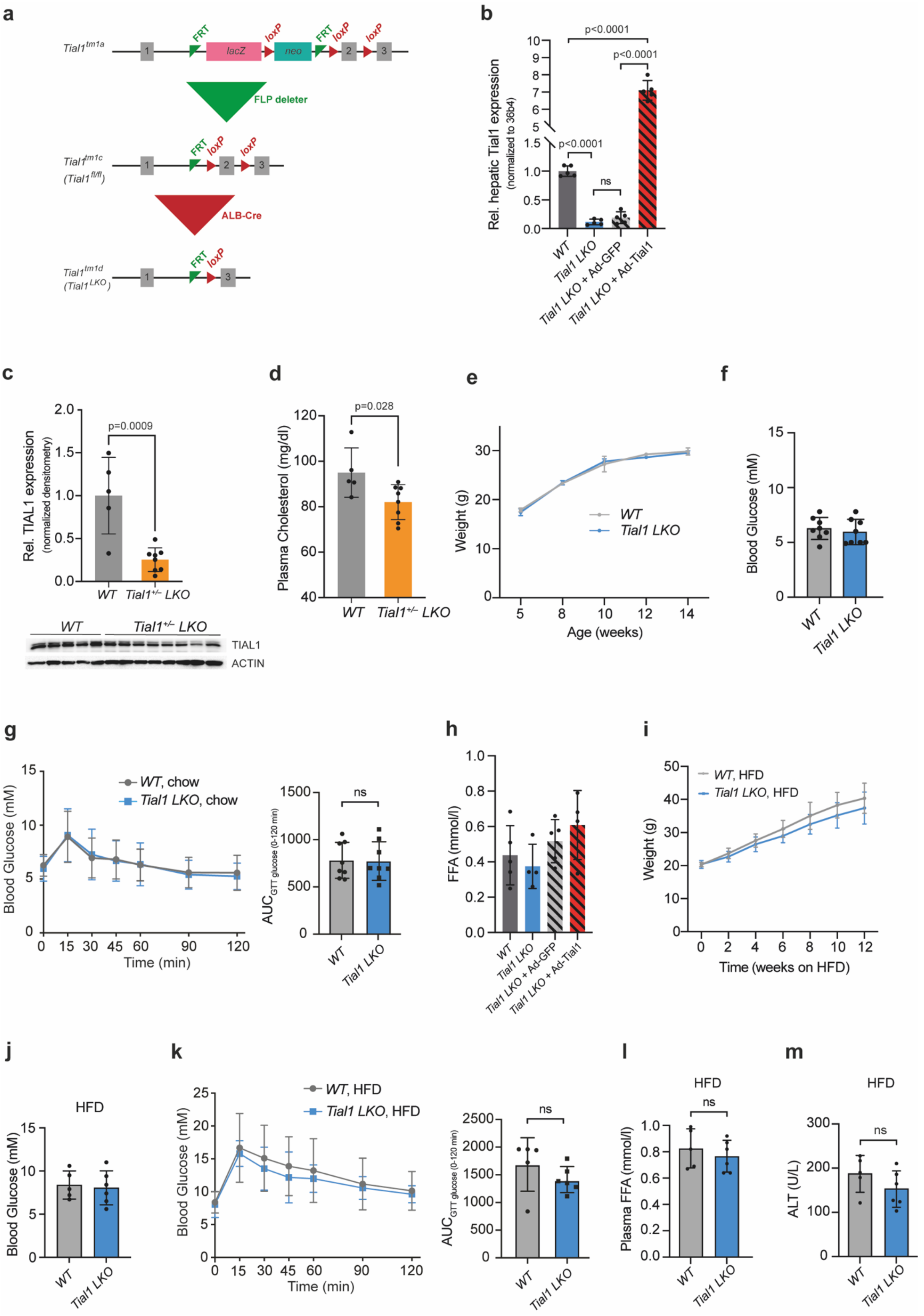

Supplementary Figure 3: Genetic mutations in Tial1 affect hepatic lipid metabolism. ***(continued on next page)***

Supplementary Figure 3: Genetic mutations in *Tial1* affect hepatic lipid metabolism.

(**a**) Schematic representation of the *Tial1* “knockout-first” conditional allele (tm1a) and the strategy to liver specific *Tial1* ablation.

(**b**) Relative mRNA expression of *Tial1* in livers of wildtype (WT), Tial1 LKO and Ad-Tial1 injected Tial1 LKO animals (n=5 per group). Values are normalized to 36b4.

(**c**) Relative protein expression of TIAL1 in livers of WT and heterozygous Tial1 LKO animals. Values are relative densitometric readouts normalized to ACTIN (WT n=5, *Tial1*$^{+/-}$ LKO n=8).

(**d**) Plasma total cholesterol of WT and Tial1 LKO mice (WT n=5, *Tial1*$^{+/-}$ LKO n=8).

(**e**) Bodyweight measurement of WT and Tial1 LKO mice (n=5).

(**f,g**) Blood glucose measurements (after a 6h fast) and (g) ipGTT of wildtype (WT) and Tial1 LKO mice fed a chow diet.

(**h**) Non-esterified fatty acids of WT, Tial1 LKO, and Ad-Tial1 and Ad-GFP injected Tial1 LKO animals on a chow diet (n=5 per group).

(**i–m**) Bodyweight, (j) blood glucose measurements (after a 6 h fast), (k) ipGTT, (l) plasma FFA, and (m) plasma ALT activity of wildtype (WT) and Tial1 LKO mice fed a high-fat diet (HFD). Chow diet: WT n=8, Tial1 LKO n=8; HFD: WT n=5, Tial1 LKO n=6.

Data are represented as mean values ± SDs. Statistical significance was evaluated by two-tailed Student's *t*-test (c, d, f, j, l, m) or one-way ANOVA with Holm- Šídâk post hoc analysis (b, h), or two-way ANOVA with Holm-Šídâk post hoc analysis (e, g, i, k). Source data are provided as a Source Data file.

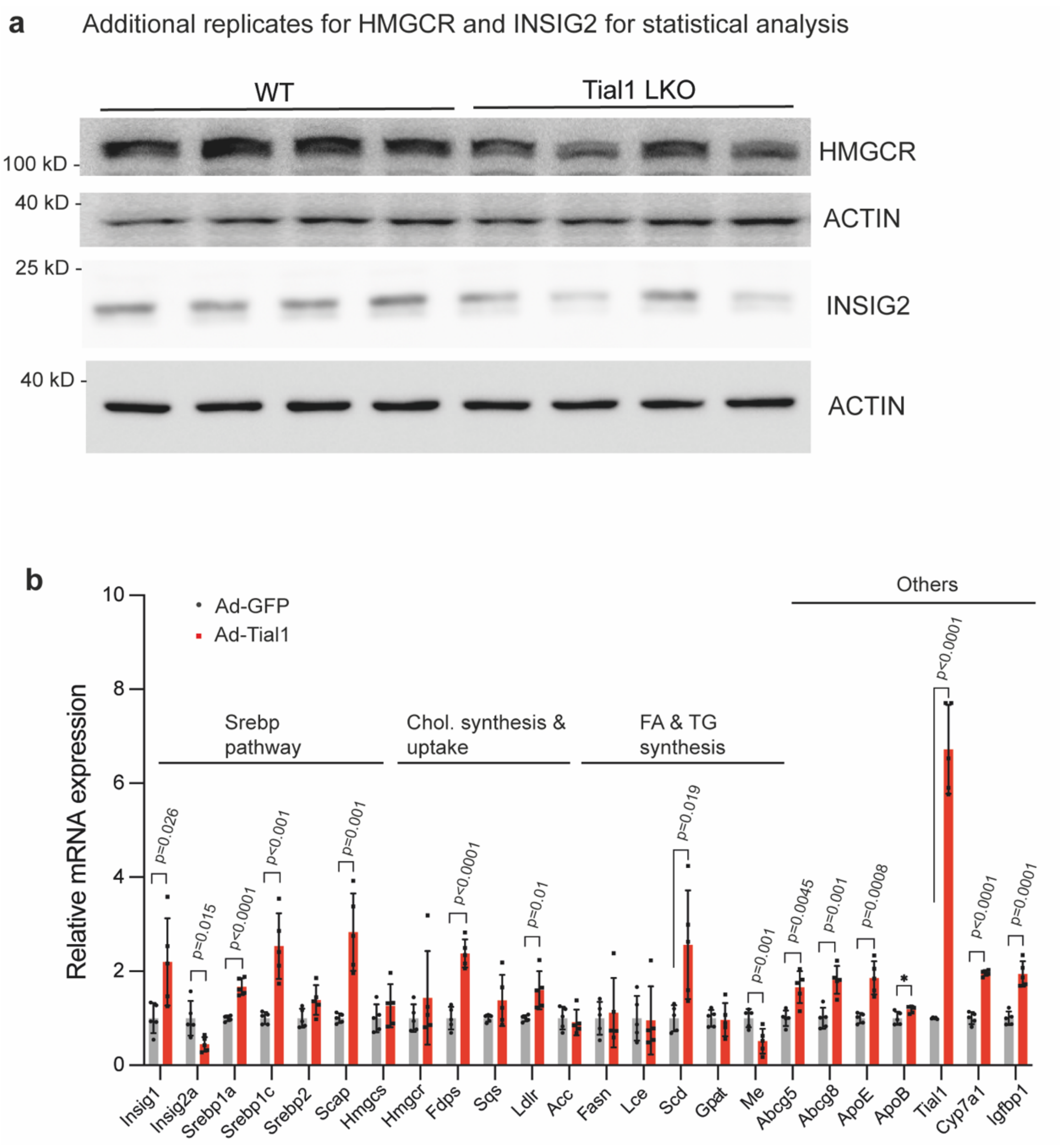

Supplementary Figure 4: Hepatic gene expression in livers of Tial1 deficient mice.

(a) Western blot analysis of HMGCR and INSIG2 in the livers of WT and Tial1 LKO mice (n=4 per group). Used as additional replicates for Fig. 4c.

(b) qRT-PCR analysis of livers of WT mice that were injected with Ad-GFP or Ad-Tial1. Shown are transcripts involved in Srebp pathway, cholesterol synthesis and uptake, and fatty acid synthesis (n=5 per group).

Data are presented as mean values ± SDs. Statistical significance was evaluated by two-tailed Student's *t*-test. Source data are provided as a Source Data file.

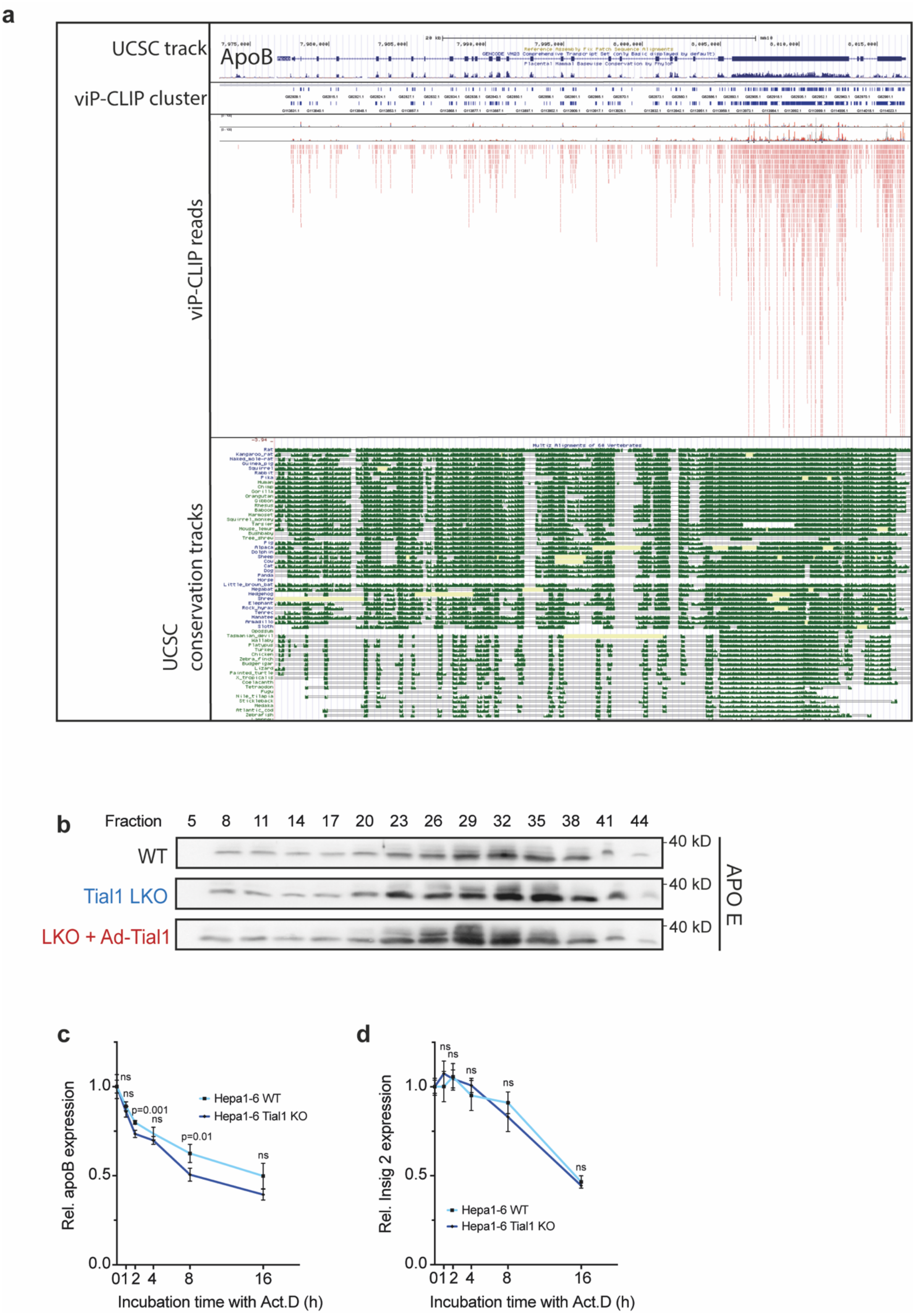

Supplementary Figure 5: TIAL1 binding sites in 3'UTR of ApoB transcript. ***(continued on next page)***

Supplementary Figure 5: TIAL1 binding sites in 3'UTR of ApoB transcript.

(a) TIAL1 liver viP-CLIP reads and cluster for ApoB mRNA, aligned to UCSC genome and conservation tracks.

(b) Immunoblot of APOE of FPLC fractions of (Fig. 5a) (n=5 mice per group).

(c, d) ApoB (c) and Insig2 (d) mRNA stability assay using transcription inhibition by actinomycin D in wildtype and Tial1 KO Hepa1-6 cells (n=4 samples per timepoint).

Data are represented as mean values ± SDs. Statistical significance was evaluated by two-tailed Student's *t*-test. Source data are provided as a Source Data file.

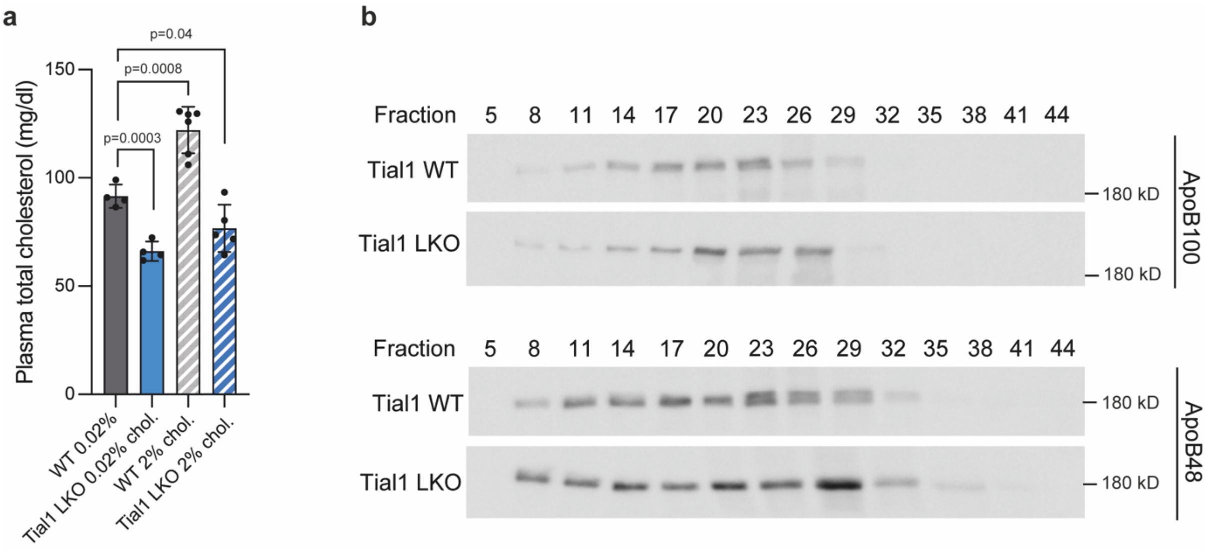

Supplementary Figure 6: Cholesterol levels in Tial1 LKO mice exposed to cholesterol rich diet.

(a) Plasma total cholesterol levels of WT and Tial1 LKO mice fed with 0.02% cholesterol (n=4) or 2% cholesterol (n=6) for 3 days ad libitum.

(b) Plasma from WT and Tial1 LKO mice fed with 2% cholesterol for 3 days ad libitum was fractionated by FPLC (n=5 per group). Indicated fractions were subjected to Western blot analysis of VLDL/IDL/LDL particles (ApoB48 bottom; ApoB100 top).

Data are represented as mean values ± SDs. Statistical significance was evaluated by one-way ANOVA with Holm-Sidâk post hoc analysis. Source data are provided as a Source Data file.

SUPPLEMENTARY TABLES

Supplementary Table 1. Primers used for qPCR

Gene	Sequence fw primer (5´ to 3´)	Sequence rv primer (5´ to 3´)
36b4	CAC TGG TCT AGG ACC CGA GAA G	GGT GCC TCT GGA GAT TTT CG
Abcg5	TGG ATC CAA CAC CTC TAT GCT AAA	GGC AGG TTT TCT CGA TGA ACT G
Abcg8	TGC CCA CCT TCC ACA TGT C	ATG AAG CCG GCA GTA AGG TAG A
Acc	TGA CAG ACT GAT CGC AGA GAA AG	TGG AGA GCC CCA CAC ACA
ApoB	CGT GGG CTC CAG CAT TCT A	TCA CCA GTC ATT TCT GCC TTT G
ApoE	GCT GGG TGC AGA CGC TTT	TGC CGT CAG TTC TTG TGT GAC T
Cyclophilin	TGG AGA GCA CCA AGA CAG ACA	TGC CGG AGT CGA CAA TGA T
Cyp7a1	GGGATTGCTGTGGTAGTGAGC	GGTATGGAATCAACCCGTTGTC
Fasn	GCT GCG GAA ACT TCA GGA AAT	AGA GAC GTG TCA CTC CTG GAC TT
Fdps	ATG GAG ATG GGC GAG TTC TTC	CCG ACC TTT CCC GTC ACA
Gpat	CAA CAC CAT CCC CGA CAT C	GTG ACC TTC GAT TAT GCG ATC A
Hmgcr	CTT GTG GAA TGC CTT GTG ATT G	AGC CGA AGC AGC ACA TGA T
Hmgcs	GCC GTG AAC TGG GTC GAA	GCA TAT ATA GCA ATG TCT CCT GCA A
Igfbp1	GAC CTC AAG AAA TGG AAG GA	GAT GTC TCA CAC TGT TTG CT
Insig 2a	CCC TCA ATG AAT GTA CTG AAG GAT T	TGT GAA GTG AAG CAG ACC AAT GT
Insig1	TCA CAG TGA CTG AGC TTC AGC A	TCA TCT TCA TCA CAC CCA GGA C
Insig2b	CCG GGC AGA GCT CAG GAT	GAA GCA GAC CAA TGT TTC AAT GG
Lce	TGT ACG CTG CCT TTA TCT TTG G-	GCG GCT TCC GAA GTT CAA
Ldlr	AGG CTG TGG GCT CCA TAG G	TGC GGT CCA GGG TCA TCT
Lxr-alpha	TCA ACC CCA TCT TCG AGT TC	ACG ACT ACT TTG ACC ACT CG
Me	GCC GGC TCT ATC CTC CTT TG	TTT GTA TGC ATC TTG CAC AAT CTT T
Scap	ATT TGC TCA CCG TGG AGA TGT T	GAA GTC ATC CAG GCC ACT ACT AAT G
Sqs	CCA ACT CAA TGG GTC TGT TCC T	TGG CTT AGC AAA GTC TTC CAA CT
Srebp1a	GGC CGA GAT GTG CGA ACT	TTG TTG ATG AGC TGG AGC ATG T
Srebp1c	GGA GCC ATG GAT TGC ACA TT	GGC CCG GGA AGT CAC TGT
Srebp2	GCG TTC TGG AGA CCA TGG A	ACA AAG TTG CTC TGA AAA CAA ATC A
Tial1	GCC CCG GAC TCT ATA CGT AGG	CAG CAG CTG CAT CTC TGT GT
Vldlr	GGCAGCAGGCAATGCAATG	GGGCTCGTCACTCCAGTCT

Supplementary Table 2. Probes used for FISH

Gene	Sequence (5' to 3')
APOB_human_1	caggagggcagaaatgatgc
APOB_human_2	acggtaaagtgagtggagca
APOB_human_3	cctttgatgagagcaagtgg
APOB_human_4	aagaagcggctgttgatctt
APOB_human_5	ctttggaggtgatgtggatt
APOB_human_6	tagtttttcagttcctgga
APOB_human_7	tcaatcagctgtggcaagag
APOB_human_8	acactgaaccaaggcttgta
APOB_human_9	acatctatcagaagggggtt
APOB_human_10	ggtaattagcaatgtccagc
APOB_human_11	agttctggagttaactgctc
APOB_human_12	atcagtgatggctttgtact
APOB_human_13	tgaagaagaacctcctggtc
APOB_human_14	tgggaagccacaaagttctt
APOB_human_15	ctgaagtccatgacagttgg
APOB_human_16	gatgaggtcagctgaagcaa
APOB_human_17	tgggaaaaatccttgcttcc
APOB_human_18	ttggtatagccaaagtggtc
APOB_human_19	attccatttaccatatcctg
APOB_human_20	cacaaactccacagacacgg
APOB_human_21	cagcttttagggcaacatga
APOB_human_22	ctgaggtgcagtaattcagg
APOB_human_23	aggttgcgctgacagaatac
APOB_human_24	tgtagcagatgagtccattt

Supplementary Table 2. Probes used for FISH (*continued*)

Gene	Sequence (5' to 3')
APOB_human_26	ggtataaggaagactcccag
APOB_human_27	ttgccaccaaaaggcaaagg
APOB_human_28	gaactctcgagatggcagat
APOB_human_29	agacccatcacatgatagtg
APOB_human_30	tctgacaagacaggccatat
APOB_human_31	catcttcatatcttcctgtt
APOB_human_32	taatttcatagatgccccag
APOB_human_33	gaatcatggcctgataagca
APOB_human_34	gagagcttaagtccttcttg
APOB_human_35	ttcatttcagcatatgagcc
APOB_human_36	gtgataagcctgcaatgttc
APOB_human_37	gctgataaggcagcagaaga
APOB_human_38	agcaacagtgtctgctttat
APOB_human_39	cgatggtcatggtaaacggg
APOB_human_40	aggtttctctgtacgttttc
APOB_human_41	ttgtgagagcagtcagtttc
APOB_human_42	gttgttttaactttccagct
APOB_human_43	catggactttggctctgaag
APOB_human_44	ctacttcatacctctcgatt
APOB_human_45	ttgtaggacattgcttagct
APOB_human_46	gccttggtttcctctaaaaa
APOB_human_47	acaggtatcgttgaagttcc
APOB_human_48	atacgtttagcccaatcttg

Supplementary Methods

A step-by-step protocol for *in vivo* Photoactivatable Ribonucleoside-Enhanced Crosslinking and Immunoprecipitation (viP-CLIP) to identify tissue specific transcriptome-wide binding sites of RNA-binding proteins in mice

Abstract

Here, we describe viP-CLIP (*in vivo* PAR-CLIP) that is capable to identify endogenous RBP targets in mammalian tissues, thereby facilitating the functional analysis of RBP-regulatory networks *in vivo*. Briefly, mice are exposed to 4-Thiouridine that is incorporated into newly transcribed RNAs in the organism, enabling an efficient UV-crosslinking in multiple tissues crucial for a successful experiment. We report a comprehensive protocol of the critical steps to adapt the cell culture PAR-CLIP[1] protocol to the viP-CLIP protocol carried out in mice.

1. Introduction:

Post-transcriptional gene regulation (PTGR) by RNA binding proteins (RBPs) are central to many biological functions, and emerging knowledge of metabolically active and insulin-sensitive organs, such as the liver, is rapidly expanding our knowledge and the scope of potential targets for treatment of metabolic diseases. The processes of the underlying pathways are mainly regulated by PTGR, which is demonstrated by the poor relationship of transcriptomic data versus the expressed protein pool[2,3]. The mechanism of PTGR is based on the formation of different ribonucleoprotein (RNP) complexes with RBPs at their core and involves the maturation, transport, stability and translation of coding and non-coding RNAs[4,5].

Techniques that combine UV crosslinking of RNA-RBP complexes followed by immunoprecipitation with high-throughput sequencing (CLIP) are suitable to identify RBP targets and binding sites to unravel the underlying mechanisms and functions[6]. An improved version is Photoactivatable-Ribonucleoside-Enhanced Crosslinking and Immunoprecipitation (PAR-CLIP). PAR-CLIP uses modified nucleotides with exocyclic thione groups, e.g., 4-thiouridine (4SU)[1,7]. Cells are grown in medium containing 4SU, which is then incorporated into the nascent RNA. 4SU allows to use UV 365 nm light to crosslinking the RBP-RNA interactions and results in increased RNA yield, due to the high reactivity of 4SU upon UV irradiation compared to conventional UV (254 nm) crosslinking.

Despite the growing amount of interest to characterize RNA-RBP interactions, current research efforts have studied RBPs using CLIP techniques to identify their bound transcriptome mainly in cultured cells[8], with a few exceptions such as *Caenorhabditis elegans*[9], mouse brain[10] or testis[11], *Drosophila*[12]. The common cultured cell lines are cancer-derived and typically present with mutations and chromosomal aberrations[13], leading to aberrant growth, metabolism and physiology[14]. A comprehensive study of RBPs and their binding partners in genuine cell types is urgently needed, however the lack of comprehensive RBP-target characterization in mammalian tissues and organs is largely owed to technical limitations of UV based methods. One of the hurdles of scaling this technique to tissues is due to the method used for cross-linking. Typically UV light of 254 nm is used which can only penetrate a few cell layers[15] and becomes a limiting factor in multi-cellular organisms. Therefore, RBP-mediated post-transcriptional modifications in the genuine physiological status during metabolic processes in tissues (e.g., amino acid, glucose, lipid, and cholesterol metabolism) are not readily studied.

Here we present the detailed viP-CLIP protocol, that is capable to identify RBP targets in mammalian tissues, thereby facilitating the functional analysis of RBP-regulatory networks *in vivo* (Figure 1). We highlight the major steps crucial to achieve and measure 4SU incorporation and distribution throughout the organism. Further, we provide details for efficient immunoprecipitations of crosslinked RBP-RNA complexes, a way to assess quality control of viP-CLIP and discuss challenges of the library preparation.

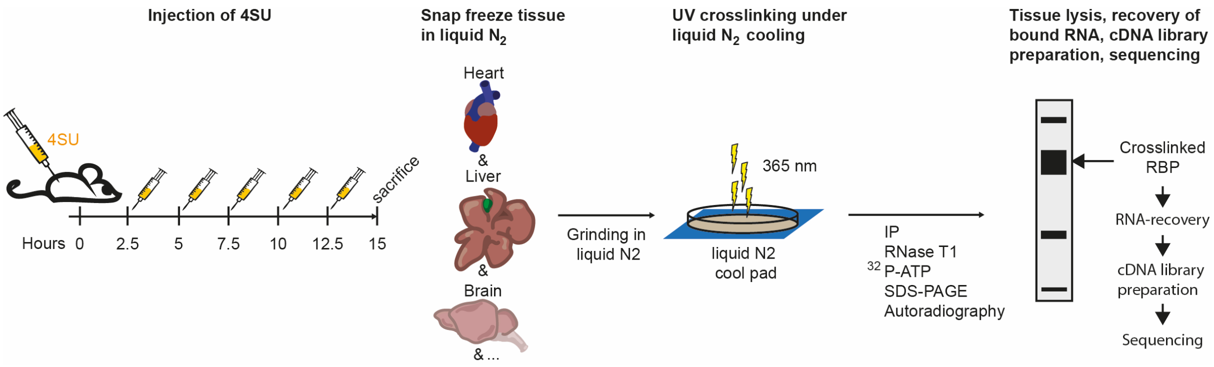

Figure 1: Schematic representation of viP-CLIP applied in mice. Mice are injected 6 times with 4SU over the time course of 12.5 h and sacrificed 15 h after the first dose. Intact organs are flash-frozen, grinded and cross-linked with UV-light (365 nm) under liquid nitrogen cooling. After cell lysis of the tissue powder, the RNA-RBP complexes are immunoprecipitated in presence of RNase, radiolabeled and the recovered RNA in the size of 19-35nt is ligated to sequencing adapter, and used for cDNA library preparation and sequencing.

2. Materials:

The following table lists viP-CLIP buffers and reagents, which differs from the optimized PAR-CLIP *Garcia et al.*[1] protocol.

Material	Composition/Notes
4-Thiouridine (powder)	Carbosytnh, NT06186-2G
PBS for injections	Invitrogen, 18912-014<br>Dissolve in Milli-Q water and autoclave.
Male C57BL/6 mice, 8-12 weeks, 25g.	Vendor: Jackson lab. Under a 12h/12h controlled photoperiod conditions, and food/water ad libitum.
NP40 lysis buffer	50 mM HEPES, pH 7.5<br>150 mM KCl<br>2 mM EDTA<br>1 mM NaF<br>1-2% (v/v) NP40, *needs to be determined before*<br>0.5 mM DTT, add freshly<br>1.5x complete EDTA-free Protease Inhibitor Cocktail (Roche), add freshly<br>1.5x Phosphatase Inhibitor Cocktail tablets PhosSTOP (Roche), add freshly<br>2x RNase Inhibitor, Murine (NEB, M0314)
NP40 dilution-buffer	50 mM HEPES, pH 7.5<br>150 mM KCl<br>2 mM EDTA<br>1 mM NaF<br>0.5 mM DTT, add freshly<br>1.5x complete EDTA-free Protease Inhibitor Cocktail (Roche), add freshly<br>1.5x Phosphatase Inhibitor Cocktail tablets PhosSTOP (Roche), add freshly<br>2x RNase Inhibitor, Murine
Bead wash-buffer	PBS, 0.1% Tween20
IP-wash	50 mM HEPES-KOH, pH 7.5<br>0.05% NP40<br>KCl: 150-500mM *needs to be determined before*<br>1x RNase Inhibitor, Murine
Phosphatase wash-buffer	50 mM Tris-HCl, pH 7.5<br>20 mM EGTA<br>0.05-0.5% (v/v) NP40 *needs to be determined before*

Material	Composition/Notes
NEB3 buffer	50 mM Tris-HCl, pH 7.9<br>100 mM NaCl<br>10 mM MgCl2<br>1 mM DTT
PNK buffer without DTT	50 mM Tris-HCl, pH 7.5<br>50 mM NaCl<br>10 mM MgCl2

3. Procedure

3.1 Preparation and harvesting of 4SU incorporated mouse tissues

1. For tagged proteins expressed by exogenous delivery, start by administration of the expression system into the mice to achieve the needed expression value by the day of 4SU administration. Routinely, we use adenovirus-mediated gene delivery to specifically target hepatocytes. Therefore, we intravenously inject $3x10^9$ PFU in a total volume of 200 µL PBS into mice, 7-10 days prior to the experiment.

2. Prepare a fresh solution of 4SU **(NOTE 1)** in PBS (injection grade) corresponding to a dose of 780 mg/kg per mouse in a total volume of 200 µL. After the 4SU has dissolved, sterile filter the solution and store in the dark at 4 °C. Use this solution within the next 24-48 hours.

3. Administer 200 µL of the 4SU solution, that has been warmed up to room temperature, by intraperitoneal injections **(NOTE 2).** The number of animals depends on the tissue specific RNA binding protein expression, its target abundance, crosslink efficiency and tissue of interest. We routinely use 5 animals and combine the organs to bigger batches.

4. Inject the solution every 2.5 hours **(NOTE 3)**, over the time-course of 12.5h. After the last injection wait 2.5 hours before scarifying the mice, so that the mice are exposed to 4SU for a total of 15 hours **(NOTE 4).**

5. Euthanize the mice using carbon dioxide (CO_2) until breathing stops. Next, transcardially perfuse the mice with ice-cold PBS. Excise the organs rapidly and immediately snap-freeze the tissue in liquid nitrogen. If studying organs with a high content of ribonucleases and proteinases, e.g., the pancreas, start dissecting these organs first.

6. Once frozen in liquid nitrogen, the tissues can be stored indefinite or at least 12 months at -80°C.

3.2 Tissue grinding and UV365-Crosslinking

1. Pre-cool the tissue grinder (Figure 2A), spatula, razorblade and crosslinking-pad with liquid nitrogen.

2. Remove the tissue from -80 °C and cool down further with liquid nitrogen.

3. Once the temperature is reached, add the tissue into the grinder and produce a fine powder with several strokes and by mixing the powder with the spatula between the strokes. It can be necessary to cool down the tissue grinder between these steps. Repeat the steps until a homogeneous fine powder is reached.

4. Next, spread the powder as a fine layer onto the liquid nitrogen cool-pad (Figure 2B) and crosslink it four times with 0.300 J/cm^{-2} at 365 nm (**NOTE 5)**. Mix the powder with the help of a razorblade between each irradiation and make sure the cooling is secured.

5. Collect the crosslinked tissue powder into a pre-cooled 50 mL falcon and snap freeze it in liquid nitrogen. The crosslinked powder can be stored indefinite or at least 12 months at -80 °C.

A B

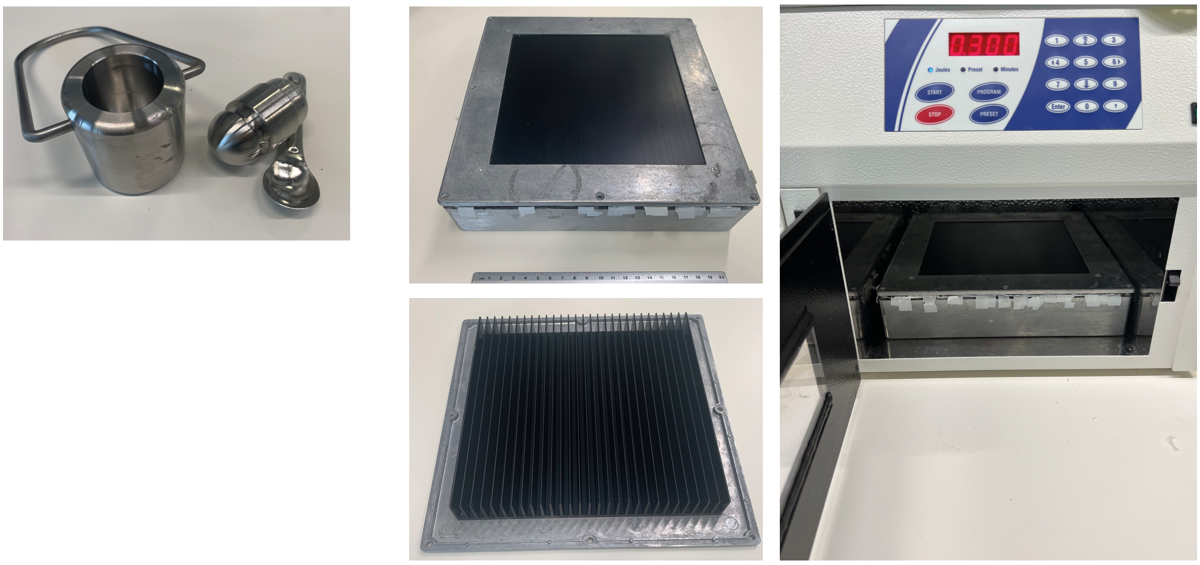

Figure 2: A. Tissue grinder; CellCrusher®. Can be used to pulverize samples from 5 mg to 10 g under liquid nitrogen cooling. The pulverized sample is recovered with an exactly fitting spoon, minimizing sample loss. **B.** Homemade liquid nitrogen cooling pad. Build out of an insulated container (grey) that is filled up with liquid nitrogen and an aluminum heatsink (black) that immerses into the liquid nitrogen, providing a cold surface for the tissue powder.

3.3 Tissue lysis and RNase treatment

1. Homogenize the tissue powder (**NOTE 6)** in a minimum of ice-cold high percentage NP40 buffer using a glass douncer with 20 strokes in an ice bath.

2. Incubate the lysate on a rotation wheel at 4 °C for 1-2 hours (start preparing the beads during this incubation time).

3. After incubation, sonicate the lysate in a Bioruptor-Plus with the setting “low” for 5 cycles 30sec on/off time (**NOTE 7)**.

4. Dilute the lysate with dilution buffer to adjust the NP40 concentration according to the antibody-antigen recognition requirements and to reach a tissue lysis buffer ratio of 1:10 **(NOTE 8)** and Figure 3.

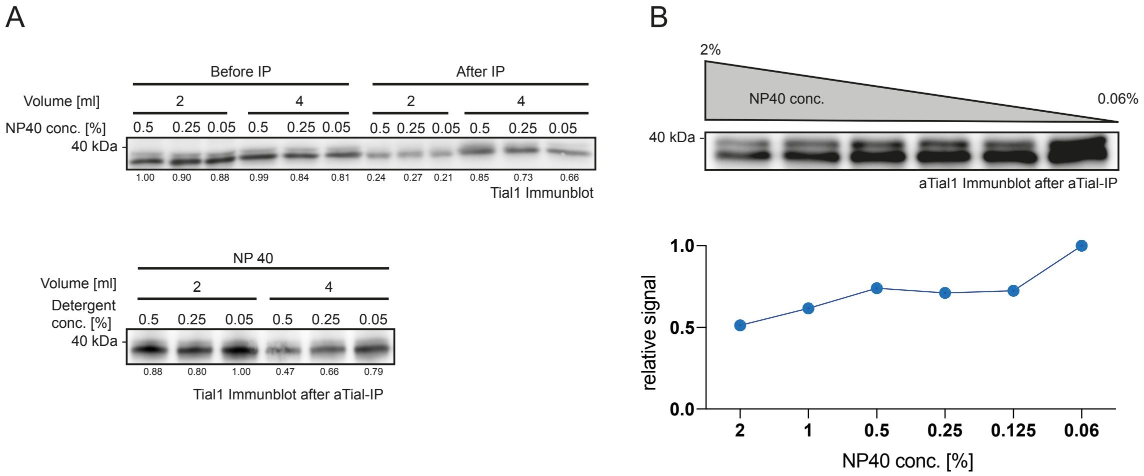

Figure 3: Detergent concentration is crucial for an efficient tissue lysis and subsequent immunoprecipitation. **A.** Immunoblot of liver tissue lysate for Tial1 viP-CLIP, displaying that during the lysis a high NP40 concentration is crucial for efficient lysis and recovery of Tial1 from liver while keeping the volume and dilution of the sample low. During IP dilution of NP40 increases the Tial1 IP. The relative densitometry result is indicated as numbers under the detected bands. **B.** Immunoblot of liver tissue lysate for Tial1 viP-CLIP with decreasing amount of NP40, displaying the increased immunoprecipitation of Tial1 in low detergent concentrations. The relative densitometry of the immunoblot signals are shown in the graph below. Representative experiments of three independent replicates (for A and B).

5. Clear the lysate by centrifugation at 13,000 g at 4 °C for 30 min and filter through a 0.45 µm membrane filter (low protein binding filter). If the tissue has a high amount of fat, it can be necessary to remove the fat layer before filtration.

6. For an efficient lysis and recovery of RNAs in the fragment size of 20-40 nt, add the upfront determined concentration of RNaseT1 to the lysate (**NOTE 9)** and Figure 4.

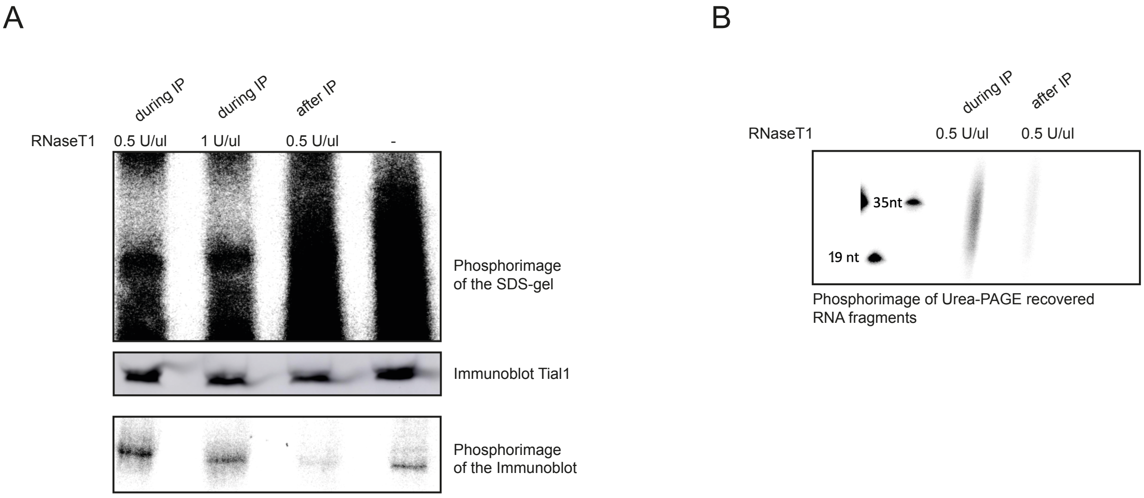

Figure 4: IP efficiency of crosslinked RBP-RNA is highly dependent on RNase treatment during the IP. **A.** Tial1 liver viP-CLIP displaying the increased IP efficiency of crosslinked RNA if RNase is added during the overnight-incubation compared to the RNA digestion after the overnight incubation. **B.** Recovered RNAs of the sampled in A. with an increased amount of crosslinked RNA in the sample subjected to overnight digestion (during IP) versus the digestion (after IP). Representative experiments of three independent replicates (for A and B).

3.4 Bead preparation

1. Prepare a fresh tube with 5 mg protein G magnetic beads per mL lysate.

2. Place the tube on the magnetic rack to separate the beads from the storage solution and wash the beads three times with bead wash buffer. Do not let the beads get dry at any point.

3. After the final wash remove the bead wash buffer and add 10 µg of the antibody adjusted to the original bead volume with PBS-Tween **(NOTE 10)**.

4. Incubate the beads by rotation at room temperature for 2 hours.

5. After incubation wash the beads twice with ice-cold NP40 lysis buffer that has the same final NP40 concentration as described in 3.3 point 4.

3.5 Immunoprecipitation

1. Remove the buffer from the beads prepared in 3.4 and directly add the lysate prepared in step 3.3.

2. Incubate the samples by rotating them at 4 °C overnight.

3. Collect the beads with the magnetic stand and discard or use the supernatant for subsequent immunoprecipitations with other antibodies, keeping in mind the long ribonuclease T1 incubation. If performing subsequent immunoprecipitations, add additional ribonuclease and proteinase inhibitors to the lysate. It can be necessary to also stop the ribonuclease T1 digestion at this point, by choosing an appropriate inhibitor.

4. Wash the beads three times with IP wash buffer (**NOTE 11**).

5. Wash the beads twice in NEB3 buffer and resuspend the beads in 1 volume of NEB3.

6. Add calf intestinal alkaline phosphatase (10 U/µl) to a final concentration of 0.5 U/µl, and incubate the suspension for 30 min at 37°C with shaking the beads every 2 min for 15 sec at 1200 rpm.

7. Wash the beads twice in phosphatase wash buffer.

8. Wash the beads twice in PNK buffer without DTT and resuspend the beads in 1 volume of PNK buffer with DTT and immediately continue with the radiolabeling reaction as described in the original protocol (starting from step 3.3.6) *Garcia et al.*[1] **(NOTE 12).**

All the following steps; recovery of RNA-RBP complex from nitrocellulose membrane, proteinase K digestion, RNA fragment size selection, adapter ligation, cDNA library preparation, PCR amplification, and computational data analysis are done according to the previously published protocol *Garcia et al.*[1].

NOTE:

1. Before using 4SU for *in vivo* application a quality control should be performed to ensure purity of the product. Bigger batches of 4SU are therefore subjected to ^{1}H-NMR and ^{13}C-NMR [16]. Degradation products or impurities should be removed by recrystallization or column chromatography with HPLC.

2. Make sure that this experimental protocol is approved by the animal use & care committees and in compliance with the guidelines according your institution and country location.

3. We routinely performed our experiments with C57BL/6 mice; however, it can be necessary to measure the clearance of 4SU in the blood (extended methods: Measurement of plasma 4SU pharmacokinetics via UV-VIS) to adjust the injection regimen for other mouse strains, with successively determination of incorporation rates of 4SU in the total RNA of the tissue of interest (extended methods: Measurement of 4SU incorporation rates in whole tissue total).

4. This injection regimen will capture the majority of transcript, that have a half-life which falls into that timeframe. It can be useful to adjust the time for transcripts with longer or shorter half-lives. For short-lived transcripts, reduce the time between the last injection and time of sacrifice.

5. For each new tissue and RBP the crosslinking should be optimized. Therefore, crosslink the tissue with increasing energy and choose the energy setting right under the crosslink saturation point. Prolonged exposure to UV light will produce undesirable RNA phosphodiester backbone breaks or RNA-RNA inter- and intrastrand crosslinks.

6. The starting amount of tissue powder depends on the RBP protein and its crosslinking ability and needs to be determined experimentally. For large scale Tial1 viP-CLIP in liver we usually start with 3 g.

7. For an efficient sonication, do not exceed the maximum volume of lysate per tube as indicated in the instruction manual. E.g., 1.5 mL Eppendorf tubes shouldn't be filled up with more than 300 µL.

8. A high NP40 concentration of >1% is necessary to efficiently capture nuclear interaction e.g., for RBPs with intronic binding affinity. However, the high concentration of detergent can be detrimental for the antibody-antigen recognition and needs to be determined before proceeding with the viP-CLIP protocol.

9. Omitting the ribonuclease treatment during the IP largely captures RBPs that are less bound to RNA or with a bias to shorter RNAs, as indicated by the same amount of immunoprecipitated proteins (shown in Figure 4). However, the amount of crosslinked RNA decreases as the IP of large RBP-RNA complexes is difficult to immunoprecipitated. Therefore, the ribonuclease treatment should always be performed during the incubation with the beads.

10. Protein G Dynabeads: check the binding affinity and capacity of the beads to the specific antibody used and change the ratio of bead to antibody according to the manufacturer notes. We routinely couple 10 µg antibody to 5 mg protein G magnetic beads.

11. Depending on the magnetic-beads and conjugated antibody, the ionic strength of the salt concentration can interfere with the binding ability of the antibody. Therefore, the salt concentration of the IP-wash and the high-salt wash buffer needs to be determined before conducting the experiment.

12. For viP-CLIP we routinely blot the RBP-RNA complexes to nitrocellulose. This helps to detect RBPs with lower crosslinking signals and removes non-crosslinked background RNAs that migrate together with the RBP-RNA in the SDS-PAGE gel.

4. Additional considerations

4.1 Tissue specific viP-CLIP targets

When performing viP-CLIP for a RBP in multiple tissues, a reasonable indicator of successful experiments is to assess if the identified targets are tissue specific (Figure 5). This kind of quality control should be done before normalizing the viP-CLIP data for the tissue specific transcriptome.

A

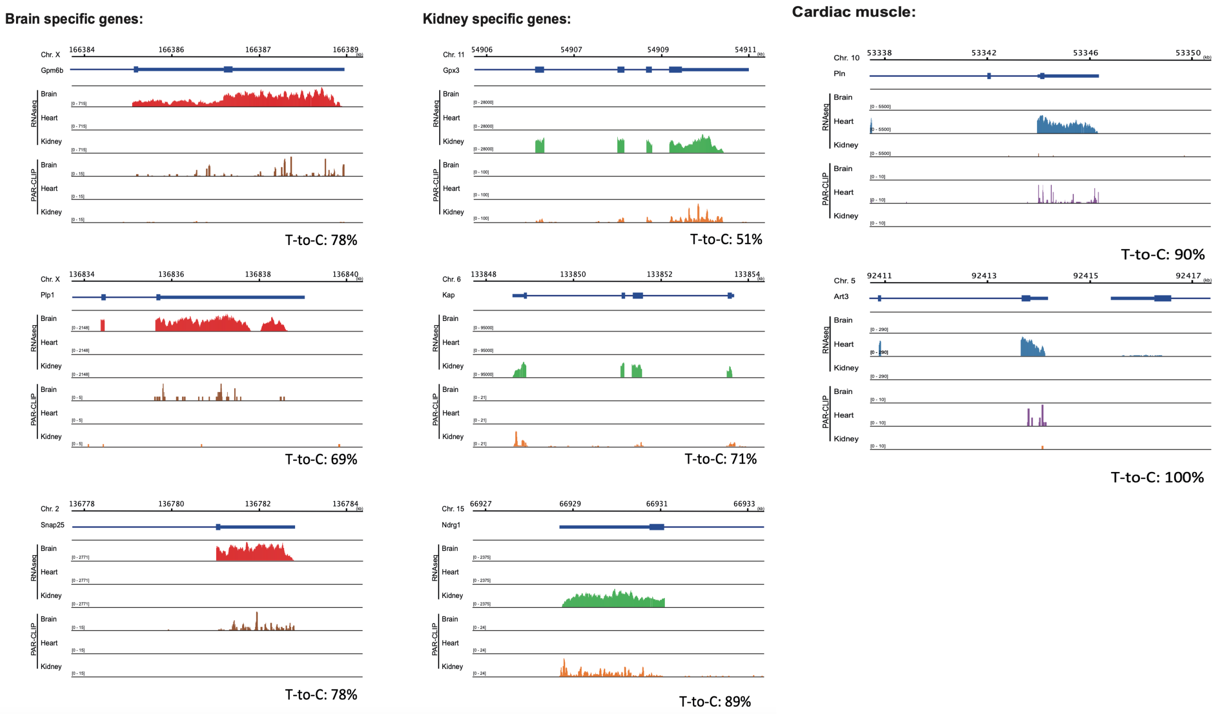

Figure 5: Alignment of Tial1 viP-CLIP in Brain, Kidney and Heart with transcriptomic data. As indicated, the viP-CLIP identified and annotated reads in tissue specific targets are not found in other unrelated tissues.

4.2 Library preparation

A major bottle-neck in viP-CLIP, as well as other CLIP approaches, is the library preparation step. After proteinase K digestion a short polypeptide remains at the nucleotide that was crosslinked to the RPB, leading to stalling and termination of the reverse transcriptase in over 80% of cases[17]. We investigated if we can overcome this obstacle by using other reverse transcriptase enzymes, but we didn't succeed. Further, we tested an approach using blocking antisense-oligonucleotides (Figure 6A) against the 3'adapter to avoid the formation of adapter-adapter dimers and adding Poly-ethylene-glycol (PEG) to enhance the reverse transcriptase reaction. Both approaches alone and in combination were promising, when assessing the cDNA products of Tial1 PAR-CLIP in HEK293 and viP-CLIP in liver on agarose gels. We could detect increased signals in the size according to a successful library preparation (Figure 6B). However, sequencing revealed that the blocking oligo and the PEG approach led to strong biases. The blocking oligo led to a shorter artificial sequence that took up the majority of sequencing reads. PEG increased the efficiency mainly for shorter reads shifting the whole read distribution in to a short fragment window, which are too short for the annotation against the transcriptome (Figure 6C).

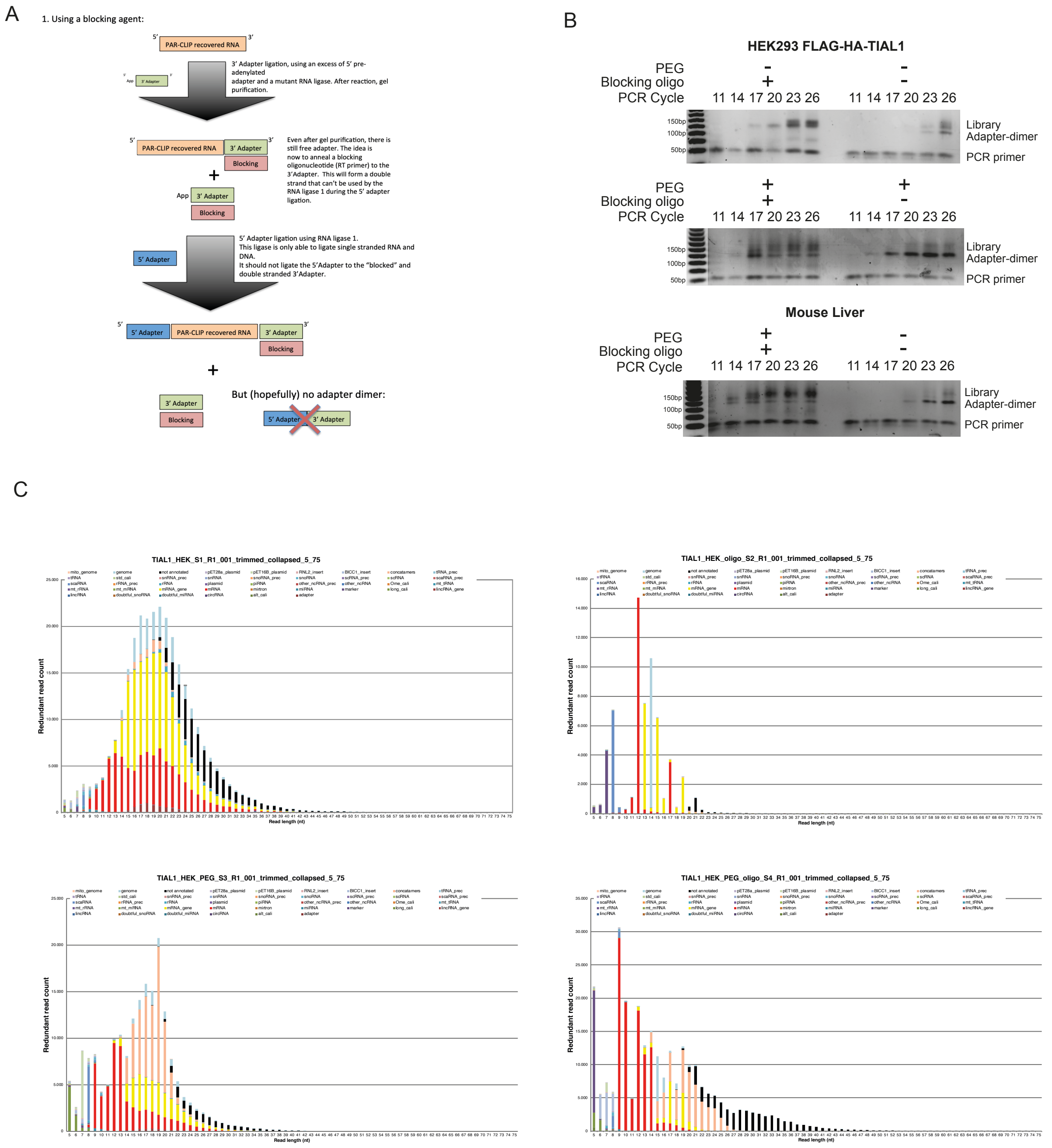

Figure 6: Approaches to increase library preparation by optimization of the reverse transcriptase reaction. **A.** Schematic of using blocking oligos to avoid adapter-dimer formation. **B.** Agarose gel of Tial1 PAR-CLIP in HEK293 and Tial1 liver viP-CLIP using blocking oligo and PEG in different variations. Representative experiment of three independent replicates. **C.** Sequencing read distribution of the libraries prepared from (B).

5. Conclusion

PAR-CLIP has mainly been applied to study the RBP-RNA interaction of more than 100 RBPs in cell culture experiments. Although there have been continuous improvements of the method, none have been addressed its execution in *in vivo*

models. To contribute to the development of the technique and its application in mice models, we provided our detailed changes leading to the viP-CLIP protocol. As a proof of principle and case study of our developed *in vivo* PAR-CLIP approach, we employed our method to study the hepatic function of two RBPs, A1cf[18] and Tial1 (in submission). Our protocol will help to comprehensively identify tissue specific RBP targets, binding sites and functions in the nearer future.

6. Extended methods:

Measurement of plasma 4SU pharmacokinetics via UV-VIS:

1. Prepare a fresh serial dilution of known 4SU concentration in PBS that lies in between the minimum and maximum absorbance range of the UV spectroscope. We routinely use the concentration range of 0-1 mM 4SU. Measure the appropriate absorbance values at 330 nm and calculate a standard curve.

2. Measure the plasma 4SU concentration at several timepoints after injection. We routinely follow the plasma distribution and clearance every 20 minutes for 180 minutes. Depending on the spectroscope, it can be necessary to dilute the sample with PBS to get values within the linear range.

3. Each sample and standard should be measured in triplicates, and the concentration of the sample will be calculated using the standard curve.

   Note: If using any kind of additional drug treatment, make sure that the drug does not interfere with the UV-VIS spectrum by recording the absorption-

spectra between 200-400 nm of blood samples of treated versus untreated animals.

Measurement of 4SU incorporation rates in whole tissue total RNA:

Adapted from Spitzer et al.[19]

1. Frozen tissue powder (starting amount e.g.,20 mg) from step 3.2.3 is homogenized in TRIzol, 20 µL per mg tissue, using a glass homogenizer and 30 strokes on ice. It is important to use not crosslinked tissue powder.
2. Remove the tissue fat layer by centrifugation with 13,000 g at 4 °C for 10 min and carefully pipetting the clear lysate under the fat layer to a new, pre-cooled tube.
3. Proceed with the protocol according to the manufacturer's instructions.
4. To prevent oxidization of the thiocarbonyl group, all subsequent isopropanol and ethanol washing steps include 0.1 mM Dithiothreitol.
5. The RNA pellet is dissolved in 25 µL H_2O and the concentration is determined.
6. For the digestion reaction set up 3 tubes. One containing 20-40 µg of the recovered tissue RNA, the other two are synthetic control RNAs with and without 4SU.
7. Perform each reaction in a total of 30 µL H_2O containing 10 mM $MgCl_2$, 100 mM Tris-HCl (pH 7.5), 0.5 U Calf Intestinal Alkaline Phosphatase (CIP) and 0.1 U Snake Venom Phosphodiesterase (SVP).
8. Incubate over night at 37°C.
9. The reaction is stopped and cleared using centrifugal filters, and can be stored at -20°C until measurement.

10. The single ribonucleotides are separated via HPLC on a Supelco Discovery C18 (bonded phase silica 5 µM particle, 250 x 4.6 mm) reverse phase column (Sigma).
11. Perform the separation with an isocratic gradient: 0 % B for 15 min, 0 to 10 % B for 20 min, 10 to 100% B for 30 min, mobile phase (A) 3 % acetonitrile in 0.1 M TEAA (92:5:3 deionized water: 2 M TEAA: acetonitrile) and mobile phase (B) 90 % acetonitrile in water.
12. The HPLC column was cleaned between the runs with 100 % (B).
13. Retention time of Uridine and 4SU is confirmed by co-injection of the standards.
14. 4SU substitution rates are calculated with the help of the absorbance rate of the synthetic 4SU containing ctrl sample and by diving the area under the curve by the extinction coefficients of Uridine at 260 nm versus 4SU at 330 nm.

Supplementary References

1 Garzia, A., Meyer, C., Morozov, P., Sajek, M. & Tuschl, T. Optimization of PAR-CLIP for transcriptome-wide identification of binding sites of RNA-binding proteins. *Methods* **118-119**, 24-40, doi:10.1016/j.ymeth.2016.10.007 (2017).

2 de Sousa Abreu, R., Penalva, L. O., Marcotte, E. M. & Vogel, C. Global signatures of protein and mRNA expression levels. *Mol Biosyst* **5**, 1512-1526, doi:10.1039/b908315d (2009).

3 Vogel, C. *et al.* Sequence signatures and mRNA concentration can explain two-thirds of protein abundance variation in a human cell line. *Mol Syst Biol* **6**, 400, doi:10.1038/msb.2010.59 (2010).

4 Gerstberger, S., Hafner, M. & Tuschl, T. A census of human RNA-binding proteins. *Nat Rev Genet* **15**, 829-845, doi:10.1038/nrg3813 (2014).

5 Janga, S. C. & Mittal, N. Construction, structure and dynamics of post-transcriptional regulatory network directed by RNA-binding proteins. *Adv Exp Med Biol* **722**, 103-117, doi:10.1007/978-1-4614-0332-6_7 (2011).

6 Wang, T. *et al.* Design and bioinformatics analysis of genome-wide CLIP experiments. *Nucleic Acids Res* **43**, 5263-5274, doi:10.1093/nar/gkv439 (2015).

7 Hafner, M. *et al.* Transcriptome-wide identification of RNA-binding protein and microRNA target sites by PAR-CLIP. *Cell* **141**, 129-141, doi:10.1016/j.cell.2010.03.009 (2010).

8 Lee, F. C. Y. & Ule, J. Advances in CLIP Technologies for Studies of Protein-RNA Interactions. *Mol Cell* **69**, 354-369, doi:10.1016/j.molcel.2018.01.005 (2018).
9 Jungkamp, A. C. *et al.* In vivo and transcriptome-wide identification of RNA binding protein target sites. *Mol Cell* **44**, 828-840, doi:10.1016/j.molcel.2011.11.009 (2011).
10 Ule, J. *et al.* CLIP identifies Nova-regulated RNA networks in the brain. *Science* **302**, 1212-1215, doi:10.1126/science.1090095 (2003).
11 Xu, Q. *et al.* Enhanced Crosslinking Immunoprecipitation (eCLIP) Method for Efficient Identification of Protein-bound RNA in Mouse Testis. *J Vis Exp*, doi:10.3791/59681 (2019).
12 Hansen, H. T. *et al.* Drosophila Imp iCLIP identifies an RNA assemblage coordinating F-actin formation. *Genome Biol* **16**, 123, doi:10.1186/s13059-015-0687-0 (2015).
13 Leung, E., Kim, J. E., Askarian-Amiri, M., Finlay, G. J. & Baguley, B. C. Evidence for the existence of triple-negative variants in the MCF-7 breast cancer cell population. *Biomed Res Int* **2014**, 836769, doi:10.1155/2014/836769 (2014).
14 Ben-David, U. *et al.* Genetic and transcriptional evolution alters cancer cell line drug response. *Nature* **560**, 325-330, doi:10.1038/s41586-018-0409-3 (2018).
15 Nierzwicki-Bauer, S. A., Gebhardt, J. S., Linkkila, L. & Walsh, K. A comparison of UV cross-linking and vacuum baking for nucleic acid immobilization and retention. *Biotechniques* **9**, 472-478 (1990).
16 Moreno, S. *et al.* Synthesis of 4-thiouridines with prodrug functionalization for RNA metabolic labeling. *RSC Chem Biol* **3**, 447-455, doi:10.1039/d2cb00001f (2022).
17 Huppertz, I. *et al.* iCLIP: protein-RNA interactions at nucleotide resolution. *Methods* **65**, 274-287, doi:10.1016/j.ymeth.2013.10.011 (2014).
18 Nikolaou, K. C. *et al.* The RNA-Binding Protein A1CF Regulates Hepatic Fructose and Glycerol Metabolism via Alternative RNA Splicing. *Cell Rep* **29**, 283-300 e288, doi:10.1016/j.celrep.2019.08.100 (2019).
19 Spitzer, J. *et al.* PAR-CLIP (Photoactivatable Ribonucleoside-Enhanced Crosslinking and Immunoprecipitation): a step-by-step protocol to the transcriptome-wide identification of binding sites of RNA-binding proteins. *Methods Enzymol* **539**, 113-161, doi:10.1016/B978-0-12-420120-0.00008-6 (2014).
