# Supplementary material for: In vivo PAR-CLIP (viP-CLIP) of liver TIAL1 unveils targets regulating cholesterol synthesis and secretion
Source: Nat Commun. 2023 Jun 9;14:3386. doi: 10.1038/s41467-023-39135-8 (PMC10256721; doi:10.1038/s41467-023-39135-8)
Supplement: Supplementary file 2 — Reporting Summary [file 41467_2023_39135_MOESM2_ESM.pdf]

nature portfolio

Corresponding author(s): Markus Stoffel
ETH Zurich, Otto-Stern-Weg 7, 8093 Zürich, Switzerland
e-mail: stoffel@biol.ethz.ch

Last updated by author(s): 27.05.2023

Reporting Summary

Nature Portfolio wishes to improve the reproducibility of the work that we publish. This form provides structure for consistency and transparency in reporting. For further information on Nature Portfolio policies, see our Editorial Policies and the Editorial Policy Checklist.

Statistics

For all statistical analyses, confirm that the following items are present in the figure legend, table legend, main text, or Methods section.

n/a	Confirmed	
☐	☒	The exact sample size (*n*) for each experimental group/condition, given as a discrete number and unit of measurement
☐	☒	A statement on whether measurements were taken from distinct samples or whether the same sample was measured repeatedly
☐	☒	The statistical test(s) used AND whether they are one- or two-sided<br>*Only common tests should be described solely by name; describe more complex techniques in the Methods section.*
☐	☒	A description of all covariates tested
☐	☒	A description of any assumptions or corrections, such as tests of normality and adjustment for multiple comparisons
☐	☒	A full description of the statistical parameters including central tendency (e.g. means) or other basic estimates (e.g. regression coefficient) AND variation (e.g. standard deviation) or associated estimates of uncertainty (e.g. confidence intervals)
☐	☒	For null hypothesis testing, the test statistic (e.g. *F*, *t*, *r*) with confidence intervals, effect sizes, degrees of freedom and *P* value noted<br>*Give P values as exact values whenever suitable.*
☒	☐	For Bayesian analysis, information on the choice of priors and Markov chain Monte Carlo settings
☒	☐	For hierarchical and complex designs, identification of the appropriate level for tests and full reporting of outcomes
☐	☒	Estimates of effect sizes (e.g. Cohen's *d*, Pearson's *r*), indicating how they were calculated

Our web collection on statistics for biologists contains articles on many of the points above.

Software and code

Policy information about availability of computer code

Data collection	No specific software was used.
Data analysis	For RNA sequencing analysis, raw reads were cleaned using Trimmomatic, version 0. 36.<br>Sequence pseudo alignment of the resulting high-quality reads to the mouse reference genome and quantification of gene level expression were carried out using Kallisto (Version 0.4 4). Differentially expressed genes were identified using the R package edgeR from Bioconductor, version 20.<br>Pathway enrichment analysis was performed by using Ingenuity (QIAGEN).<br>ParCLIP data were analyzed with the PAR-CLIP suite and PARalyzer software using PARalyzer version 2.0.<br>Motive analysis was performed using MEME-ChIP version 5.5.2.<br>Image quantification was performed using ImageJ or QuPath software version 0.1.2.<br>Image acquisition for FISH was performed using VisiView software, version 5.0.0.16.<br>Western Blot densitometry was performed using ImageJ version 1.53 c.<br>Statistical analysis was performed using GraphPad Prism version 9.

For manuscripts utilizing custom algorithms or software that are central to the research but not yet described in published literature, software must be made available to editors and reviewers. We strongly encourage code deposition in a community repository (e.g. GitHub). See the Nature Portfolio guidelines for submitting code & software for further information.

Data

Policy information about availability of data

All manuscripts must include a data availability statement. This statement should provide the following information, where applicable:

- Accession codes, unique identifiers, or web links for publicly available datasets
- A description of any restrictions on data availability
- For clinical datasets or third party data, please ensure that the statement adheres to our policy

Raw sequencingdata for all datatypes have been deposited into public repositories. The accession numbers for the differential expression and PAR-CLIP data have been deposited in NCBI SRA: PRJNA869588.

Research involving human participants, their data, or biological material

Policy information about studies with human participants or human data. See also policy information about sex, gender (identity/presentation), and sexual orientation and race, ethnicity and racism.

Reporting on sex and gender	No Human data was used.
Reporting on race, ethnicity, or other socially relevant groupings	NA
Population characteristics	NA
Recruitment	NA
Ethics oversight	NA

Note that full information on the approval of the study protocol must also be provided in the manuscript.

Field-specific reporting

Please select the one below that is the best fit for your research. If you are not sure, read the appropriate sections before making your selection.

☒ Life sciences ☐ Behavioural & social sciences ☐ Ecological, evolutionary & environmental sciences

For a reference copy of the document with all sections, see nature.com/documents/nr-reporting-summary-flat.pdf

Life sciences study design

All studies must disclose on these points even when the disclosure is negative.

Sample size	No sample-size calculation was performed. Sample sizes were chosen based on previous experiences with our mouse models and cell lines (Mobin et al. 2017, Nat Commun. 7:12848. doi: 10.1038/ncomms12848.; Nikolaou et al. 2019, Cell Rep. 29:283-300.e8. doi: 10.1016/ j.celrep.2019.08.100.
Data exclusions	No data were excluded from analysis except rare outliers that were two standard deviations outside of the mean.
Replication	In all experiments, one sample (n) represents one animal. For H&E staining, three primary hepatocyte slides were analyzed and reported. In cell culture experiments, one sample (n) represents one well of a multi well plate. In Western blots, samples were assayed in unicates and quantifications are representative of at least 2 experiments. All attempts at replication were successful.
Randomization	For most experiments, the breeding strategy was such that all mice in a litter were either controls or transgenic/knockouts; therefore,all mice in a litter were used for experiments. Multiple litters were combined for each cohort to achieve sufficient sample sizes. For experiments that could only include a portion of the cohort for technical reasons or for allocation to experimental groups, mice were randomized in a manner that distributed littermates evenly across groups, and to ensure that their average body weight matched that of their respective group.<br>For cell experiments, no randomization was utilized. Cells samples were separated into experimental groups by treatment. Multiple biological replicas for each experimental group were assessed in each experiment and produced comparable results.
Blinding	Investigators were blinded to group allocation during data collection, sample collection/processing, and analysis

Reporting for specific materials, systems and methods

We require information from authors about some types of materials, experimental systems and methods used in many studies. Here, indicate whether each material, system or method listed is relevant to your study. If you are not sure if a list item applies to your research, read the appropriate section before selecting a response.

April 2023

Materials & experimental systems

n/a	Involved in the study
	☒ Antibodies
	☒ Eukaryotic cell lines
☒	Palaeontology and archaeology
	☒ Animals and other organisms
☒	Clinical data
☒	Dual use research of concern
☒	Plants

Methods

n/a	Involved in the study
☒	ChIP-seq
☒	Flow cytometry
☒	MRI-based neuroimaging

Antibodies

Antibodies used

The following antibodies were used in immunoblotting: mouse anti-γ-tubulin (1:10,000) (Sigma-Aldrich, #T6557), rabbit anti-Gapdh (1:500) (Santa Cruz, #2118S), rabbit anti-HuR (1:500) (Santa Cruz, #sc-20694), rabbit anti-Histone H3 (1:5,000) (Cell Signaling, #4499S), rabbit anti-apoB (1:2,000) (Meridian, #K23300R), rabbit anti-apoE (1:2,000) (Meridian, #K23100R), rabbit anti-apoA-I (1:10,000) (Meridian, #K23500R), mouse anti-Tial1 (1:1000) (BD Biosciences, #610352), mouse anti-Myc (1:1000) (Millipore, clone 4A6, #05-724), rabbit anti-ßActin (1:5000) (Cell Signaling, #4970S), rabbit anti-Insig2 (1:250) (ProteinTech, #24766), rabbit anti-Srebp2 (1:500) (ThermoScientific, #PA5-88943), rabbit mouse-Srebp1 (1:500) (ThermoScientific, #MA516124), rabbit anti-Insig1 (1:500) (Abcam, #ab70784), rabbit anti-HMGCR (1:500) (ABclonal, #A19063), rabbit anti-Scap (1:500) (ThermoScientific, #PA5-28982), rabbit anti-HSP90 (1:1000) (Cell Signaling, #4874S). The following secondary HRP conjugated Antibodies were used in immunoblotting: Goat anti-mouse IgG-HRP (1:10000) (Calbiochem, #401253), Goat anti-rabbit IgG-HRP (1:10000) (Calbiochem, #401393).

Validation

Antibody validation is available through the manufacturers' websites.

anti-γ-tubulin (Sigma-Aldrich, #T6557): Identification of 48 kD band in WB using whole cell extract of chicken fibroblasts. Negative control: no secondary antibody

anti-Gapdh (Santa Cruz, #2118S): Immunoblotting analysis of GAPDH in transfected 293T cell lysate with GAPDH antibody at 1 µg/mL.

anti-Histone H3 (Cell Signaling, #4499S): Western blot analysis of extracts from various cell lines using anti Histone H3 identifying 15kG band.

anti-apoB (Meridian, #K23300R): Western blot analysis of serum, LDL fractions, liver and jejunum samples from mouse identifying apoB100 and apoB48 proteins at >350kD and ≈200 kD.

anti-apoE (Meridian, #K23100R): Immunoblotting analysis of brain lysates, identifying band at ≈35kD.

anti-apoA-I (Meridian, #K23500R): Identification of 28.2 kD recombinant human ApoA-I by western blotting

anti-Tial1 (BD Biosciences, #610352): Immunoblotting analysis of extracts from Jurkat cells, treated with PMA 125 ng/ml 30 min using anti-TAL-1 antibody. Negative control was treated with both anti-TAL-1 antibody and the synthesized immunogen peptide.

anti-Myc (Millipore, clone 4A6, #05-724): NIH3T3 cell lysate was probed with anti-c-Myc, proteins were visualized using a donkey anti-rabbit IgG secondary antibody conjugated to HRP and a chemiluminescence detection system.

anti-ßActin (Cell Signaling, #4970S): Western blot analysis of cell extracts from various cell lines using beta-Actin Ab.

anti-Insig2 (ProteinTech, #24766): Rat liver tissue was subjected to SDS PAGE followed by western blot with Insig2 Ab at a dilution of 1:1000 at RT identifies ≈25 kDa band.

anti-Srebp2 (ThermoScientific, #PA5-88943): Western Blot analysis of SREBP2 in extracts of various cell lines using SREBP2 polyclonal antibody at a dilution of 1:1000. A HRP Goat Anti-Rabbit IgG (H+L) secondary antibody was used at a dilution of 1:10,000.

mouse-Srebp1 (ThermoScientific, #MA516124): Western blot in mouse and human hepatocytes identified ≈68 kDa protein.

anti-Insig1 (Abcam, #ab70784): 42 kDa band in Western blot using mouse, human livers, NIH 3T3 and PC12 cells and 10µg protein per lane. Secondary ab: Goat Anti-Rabbit IgG H&L (HRP) (ab205718) at 1/50000 dilution.

anti-HMGCR (ABclonal, #A19063): ≈100 kDa band in Western blot of various cell lines and 25 µg protein per lane. Secondary ab: Goat Anti-Rabbit IgG H&L (AS014) at 1/10000 dilution. Blocking 3% nonfat dry milk in TBST.

anti-Scap (ThermoScientific, #PA5-28982): Western blot identified a 98 kDa band using anti-SCAP Polyclonal Antibody in whole cell extracts (40 µg lysate) of Hep G2, HeLa, LNCaP, MCF7, HT-29 and K-562.

anti-HSP90 (Cell Signaling, #4874S): Lysates from HeLa cells, NIH3T3 cells, human Testis and Rat Brain were probed with ab at 1:1,000 dilution in western blot and identified ≈87 kDa Proteins were visualized using a Donkey Anti-Rabbit IgG secondary antibody conjugated to HRP.

Eukaryotic cell lines

Policy information about cell lines and Sex and Gender in Research

Cell line source(s)	Hepa1-6 (ATCC CRL-1830), HEK 293T cells (Dharmacon, #HCL4517)
Authentication	Cell lines were used as received without authentication.
Mycoplasma contamination	Cell lines were not tested for mycoplasma contamination.
Commonly misidentified lines (See ICLAC register)	No commonly misidentified cell lines were used in the study.

Animals and other research organisms

Policy information about studies involving animals; ARRIVE guidelines recommended for reporting animal research, and Sex and Gender in Research

Laboratory animals	Frozen sperm of mice carrying the Tial1tm1a mutation (C57BL/6N Atm1Brd Tial1tm1a(EUCOMM)Wtsi / WtsiH) were obtained from the EMMA mouse repository (EMMA ID 09761 and used for in vitro fertilization. Briefly, the Tial1tm1a KO first allele contains a IRES: LacZ trapping cassette and a floxed promoter-driven neo cassette inserted into the second intron of Tial1 gene at position 128056386 of Chromosome 7. Tial1tm1a mice were initially crossed with Flp-1 transgenic line (Jackson Lab) to remove the FRT-flanked lacZ-neo cassette, converting the "knockout-first" allele to a conditional allele (Tial1fl/fl) (Fig. S3A). Successively, hepatocyte-specific knockout animals were obtained by crossing with Albumin-Cre transgenic mice (Jackson Lab) leading to exon 2 deletion and generation of a frameshift mutation. Heterozygous intercrosses resulted in viable and fertile homozygous Tial1 LKO (Tial1fl/fl/Alb-Cre). mice maintained on a C57BL/6N background. Mice were housed in a pathogen-free animal facility at the Institute of Molecular Health Sciences at ETH Zurich, in a temperature-controlled room (22°C), with humidity at 55% and on a 12 h light–dark cycle (lights on from 6:00 to 18:00). Mice were fed standard laboratory chow, a high-fat diet (fat, carbohydrate, protein content was 45, 35 and 20 kcal%, respectively) (Research Diets, D12451), a chow diet supplemented with 2% cholesterol, and water ad libitum. All ethical regulations were complied with, and all animal experiments were approved by the Kantonale Veterinäramt Zürich. Unless otherwise indicated in the figures and figure legends, all experiments were performed in randomly chosen age-matched male mice using littermates as controls. All WT control animals were littermates (Tial1fl/fl).
Wild animals	No wild animals were used in the study.
Reporting on sex	Male mice were used for most experiments. Key phenotypic traits observed in male mice was confirmed in female mice.
Field-collected samples	No field collected samples were used in the study.
Ethics oversight	All animal experiments were approved by the Ethics Committee of the Kantonale Veterinäramt Zürich.

Note that full information on the approval of the study protocol must also be provided in the manuscript.
